# The Role of Selenium in Pathologies: An Updated Review

**DOI:** 10.3390/antiox11020251

**Published:** 2022-01-27

**Authors:** Giulia Barchielli, Antonella Capperucci, Damiano Tanini

**Affiliations:** Dipartimento di Chimica ‘Ugo Schiff’, Università di Firenze, Via della Lastruccia 3-13, 50019 Sesto Fiorentino (Florence), Italy; giulia.barchielli1@stud.unifi.it (G.B.); antonella.capperucci@unifi.it (A.C.)

**Keywords:** selenium, antioxidants, ebselen, GPx, TrxR, inflammation, cancer, COVID-19, fertility, gender medicine

## Abstract

Selenium is an essential microelement required for a number of biological functions. Selenium—and more specifically the amino acid selenocysteine—is present in at least 25 human selenoproteins involved in a wide variety of essential biological functions, ranging from the regulation of reactive oxygen species (ROS) concentration to the biosynthesis of hormones. These processes also play a central role in preventing and modulating the clinical outcome of several diseases, including cancer, diabetes, Alzheimer’s disease, mental disorders, cardiovascular disorders, fertility impairments, inflammation, and infections (including SARS-CoV-2). Over the past years, a number of studies focusing on the relationship between selenium and such pathologies have been reported. Generally, an adequate selenium nutritional state—and in some cases selenium supplementation—have been related to improved prognostic outcome and reduced risk of developing several diseases. On the other hand, supra-nutritional levels might have adverse effects. The results of recent studies focusing on these topics are summarized and discussed in this review, with particular emphasis on advances achieved in the last decade.

## 1. Introduction

Selenium effects on human health are mainly related to the biological role of selenoproteins, even though some specific effects of different selenium-containing compounds have been described. Glutathione peroxidases (GPxs) and thioredoxin reductases (TrxRs) are involved in protection against oxidative stress, the main cause of the onset and progression of several pathologies [1,2,3].

Eukaryotes nuclear selenoproteins act in maintaining the genome against oxidative stress. Among them, SELENOH is the only selenoprotein known to be nucleus-exclusive. Healthspan deterioration may be associated with reduced expression of some selenoproteins belonging to glutathione peroxidases, thioredoxin reductases, and thioredoxin-like(Rdx) families that are particularly sensitive to a possible dietary selenium deficiency. The abovementioned selenoproteins collectively regulate redox maintenance and protein quality [4].

Some selenoproteins have been reported to paradoxically exert adverse consequences in certain contexts, including increased insulin sensitivity in GPx1 KO mice and increased cancer resistance in TrxR1-deficienct hepatocarcinoma and lung carcinoma cells. The selenium-containing GPx1, GPx2, GPx3, GPx4 are involved in the protection against oxidative stress, inhibit inflammation and oxidant-induced regulated cell death. GPx1 and GPx4 inhibit phosphorylation cascades, mainly via preventing the inactivation of phosphatases by H_2_O_2_ or lipid hydroperoxides. GPx2 regulates the balance between regeneration and apoptosis of intestinal cells and inhibits inflammation-induced carcinogenesis in the gut. However, GPx2 promotes the growth of established cancers. GPx3 deficiency facilitates platelet aggregation, probably via disinhibition of thromboxane biosynthesis pathways. GPx3 is also considered a tumour suppressor. GPx4 is expressed in three different forms: the cytosolic, the nuclear, and the mitochondrial. The cytosolic form is involved in the inhibition of interleukin-1-driven NF-κB as well as in the biosynthesis of leukotriene. It is also a key regulator of ferroptosis, because it reduces hydroperoxy groups of complex lipids and silences lipoxygenases. The nuclear form of GPx4 contributes to chromatin compaction, while the mitochondrial form is involved in the formation of the mitochondrial sheath of spermatozoa, thus playing a key role in male fertility. The expression of individual GPxs and GPx-dependent regulatory phenomena are worthy of further investigation, particularly in relation to human health [4].

Selenium has been proposed as an hormetic chemical, a compound that has a biphasic dose-response, being toxic at high doses but with beneficial attributes at very low doses. Selenium incorporation into selenoproteins reaches a plateau at nutritional levels and the remaining selenium is non-specifically incorporated in Se-containing proteins in the form of organic selenomethionine (SeMet) that replaces methionine and may eventually induce oxidative stress. Inorganic selenite and selenate are largely excreted from the body after selenoprotein expression is saturated. Low molecular weight selenium species such as free SeMet and selenols are a small portion of the total human selenium pool whose levels are quite stable in conditions ranging from selenium deficiency to excess. These non-protein forms of selenium become increasingly sizable with respect to the total pool as body selenium status decreases [5].

Selenoproteins also have a central role in nitrosative stress responses: GPxs and TrxRs metabolize nitrosothiols and peroxynitrite which are two major RNS [6]. Selenoprotein T (SELENOT) is an ER-resident protein that plays a crucial role in the maintenance of ER homeostasis, something that is pivotal in preventing proteins from misfolding and aggregating. Reduction of SELENOT expression in transgenic cells and animal models corresponded to an increased reactive oxygen and nitrogen species concentration. This leads to an accumulation of misfolded and aggregated proteins, resulting in several neurodegenerative, cardiovascular, metabolic and immune diseases [7]. Furthermore, selenoproteins influence pivotal biological functions such as Ca^2+^ signalling, spermatogenesis or brain activity [1,2,3]. SELENOT gene knockout in the brain causes anatomical alterations that impact mice behaviour, suggesting a pivotal role for brain development and function [8].

**Figure 1:**
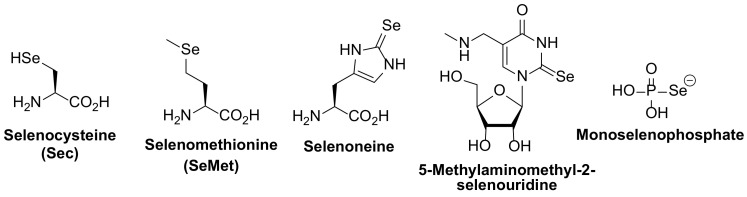
Examples of different chemical forms of selenium found in biomolecules.

Selenium is used in biology mainly in the form of selenocysteine, a true proteogenic amino acid bearing the selenol functionality (SeH), which is inserted into selenoproteins. Selenocystein is also used as a ligand for the molybdopterin guanine dinucleotide cofactor of formate dehydrogenase, for nickel in [NiFeSe] hydrogenases, as well as for iron in an iron–iron cluster. Other chemical forms of selenium used in biomolecules are depicted in Figure 1.

In a recent review entitled “Why nature chose selenium”, Reich and Hondal provided a comprehensive comparative analysis on the chemical properties of selenium and sulphur. Although selenium compounds and their sulfurated analogues share similar physical and chemical properties, including oxidation states and functional group types, there are significant differences that explain the unique biochemistry of selenoproteins.

Although the biosynthesis of selenocysteine was described as “costly” and “inefficient” [9], its incorporation into proteins enables living systems to accomplish essential chemical functions that cysteine would not be very good at. The enzymatic activity of mammalian selenoproteins including glutathione peroxidases (GPxs) [4,10], iodothyronine deiodinase [11], and thioredoxin reductases (TrxRs) [12] relies on the reactivity of the selenol moiety of a selenocysteine residue [13].

For example, the two-step mechanism accounting for the fast reactivity of GPx is based on the acidity and the nucleophilicity of the selenol moiety of the Sec residue present in the enzyme active site. The proton of the SeH group of Sec is shuttled to a tryptophan residue, highly conserved in the enzymatic pocket of all GPxs and playing the role of proton acceptor. This proton-transfer reaction leads to a high energy zwitterionic form in which the nucleophilicity of the selenolate anion is enhanced with respect to the neutral selenol [14].

The recently studied possible interactions of selenium with hydrogen sulphide further highlight the complexity of roles that selenium plays in modulating biochemical mechanisms. Hydrogen sulphide (H_2_S) belongs to a small group of metabolically active gaseous molecules called “gasotransmitters”, involved in the regulation of intracellular signalling, cellular bioenergetics, cell proliferation, and cell differentiation. It is well known that, at lower concentrations, H_2_S stimulates mitochondrial function, while at higher concentrations it suppresses the activity of mitochondrial respiratory complex IV (cytochrome C oxidase) [15,16]. Several studies have reported that H_2_S has a role in alleviating oxidative stress-induced damage from ROS in brain, gastric mucosa and hepatic ischemia-reperfusion injury, as well as vascular endothelium [17]. The three main mammalian H_2_S-generating enzymes are cystathionine-lyase (CSE), cystathionine-β-synthase (CBS), and 3-mercaptopyruvate sulfurtransferase (3-MST) [15].

In liver and intestine of animal models, a selenium deficient diet has been either associated to increased [18,19] and decreased [20] levels of H_2_S, CSE, CBS, and 3-MST in test groups with respect to control groups. H_2_S increase derived from selenium deficiency has been highlighted as the possible cause for an impaired mitochondrial-mediated apoptosis [19]. H_2_S shortage was instead associated with increased levels of the inflammatory factors TNF-α, NF-κB, COX-2, and PTGES [20]. These data suggest the existence of different mechanisms relying on an adequate selenium status, that both play a role on the proper cellular production of H_2_S, thus influencing the cellular redox homeostasis and signalling pathways.

Recently SBP1, a highly conserved protein that covalently binds selenium, has been identified as the fourth H_2_S-producer [15,21]. SBP1 has already been highlighted for playing important roles in several fundamental physiological functions, including protein degradation, cell differentiation and redox modulation, thus having a possible importance for human health and disease status [21]. SBP1 acts as a methanethiol oxidase (MTO), by converting methanethiol, an organosulfur compound from gut bacteria, into H_2_S, H_2_O_2_, and formaldehyde [16,21,22]. This finding further supported the hypothesis of a biological interplay between H_2_S and selenium (also in the form of molecules whose action depends on selenium), that has gained growing interest in recent times. SBP1 levels are frequently lower in several different cancer types with worse clinical outcome, including colorectal, gastric, nasopharyngeal, pulmonary, renal, and prostate cancers and with the only exception of ovarian cancer [16,21]. Since H_2_S have already been demonstrated to inhibit the survival of prostate cancer cells in vitro and in vivo, the reduced H_2_S levels—and thus the corresponding reduced H_2_S-mediated signalling—is associated with a lower SBP1 activity. This results in the inhibition of AMPK (an intracellular energy sensor) and stimulates the oxidative phosphorylation and the biosynthesis of building blocks needed for tumour growth and progression [16]. SBP1 was also found to have a role in the preadipocytes into adipocytes differentiation via multiple cellular signalling processes involving H_2_S [15,22]. SBP1 silencing decreased the cellular H_2_S, CBS, CSE, and 3-MST levels, and significantly suppressed adipocyte differentiation [15]. The reduced form of selenium (HSe^−^ and H_2_Se) that derives from dietary selenium shares some characteristics with H_2_S. For this reason, H_2_Se has been proposed as the fourth endogenous gasotransmitter alongside nitric oxide, carbon monoxide and hydrogen sulphide. Endogenously generated H_2_Se is indirectly incorporated into a number of selenoproteins with an oxidoreductase function. The administration of H_2_Se proved to transiently inhibit the mitochondrial cytochrome C oxidase [23,24].

CSE-derived H_2_S plays a critical role in the preservation of cardiac functions. Selenocysteine lyase (SCLY) is a homologue of cystathionine-lyase which has been identified to produce H_2_Se, the selenium homologue of H_2_S. In rat cardiac cells H9C2, H_2_S was observed to induce SCLY/H_2_Se signalling and to increase the bioavailable Se content, which then caused higher expressions and activities of selenoproteins, including glutathione peroxidase and thioredoxin reductase, followed by a reduction of ROS level and cell hypertrophy. Higher levels of H_2_S (like in the case of stressful conditions) also increase SelP levels, leading to a better distribution of selenium across the organism, especially in tissues requiring high levels of selenium for oxidative stress defence [25]. Finally, the interaction between H_2_S and SeO_3_^2−^ has been described as having several noteworthy biological effects, including ROS scavenging, modulation of the redox state, influence of blood pressure, and reaction with DNA. It may also explain the reported contrasting results on beneficial and toxic effects of selenium, for example, in conditions of oxidative stress and cancer. For this reason, H_2_S/SeO_3_^2−^ interactions should be considered when setting biological experiments with selenite, and when using it as a nutrition supplement or for clinical use [26].

An adequate selenium intake may reduce the risk of developing cancer, auto-immune diseases, sub-fertility, or mortality risk in severe illness, although some pathologies are due to specific selenoproteins genotypes [27]. In this context, in order to ensure the correct selenium intake, the use of organic or inorganic selenium supplements is a commonly employed strategy. Beyond the use of classic or novel selenium containing molecules in the form of supplements, biofortification of fruits, vegetables and crops with selenium may also represent a valuable strategy to ensure the general population an adequate selenium intake according to global or local guidelines. Selenium concentration in foodstuffs depends on the presence of available forms of this nutrient in soil, which can then be accumulated by plants and herbivorous animals. Plant biofortification strategies mainly exploit the natural metabolic pathways of plants and can be achieved through different agronomical techniques such as foliar and soil application, hydroponic conditions, or soil addition with symbiont rhizosphere microorganisms. Remarkable results have been obtained with cereal crops, vegetables, microgreens and fruit trees. The possible combination with other microelements such as iodine would afford functional vegetable food products with enhanced selenium content and general nutritional characteristic [28,29,30,31,32].

Furthermore, providing livestock with organic selenium—and in particular selenomethionine—in their feed proved to be effective in increasing the general selenium content in poultry, calves and swine meats [31]. Supplementing cows with organic selenium also increased the selenium content in milk. On the other hand, inorganic species showed limited effects [33].

However, the possibility of null or toxic rather than beneficial effects of selenium supplementation on human organism has been recently discussed [34,35,36]. Vinceti and Jablonska warn against the still uninvestigated effects of an excessive intake of selenium that may be specifically unsafe for redox homeostasis, with severe effects on the epigenetic regulation of DNA or the gut microbiota. For this reason, they even suggest a revision of the WHO RDAs for selenium [34].

Similar to other essential micronutrients, selenium plays opposite biological functions as a function of its concentration. The recommended daily allowance (RDA) of selenium is 55 µg/day; the tolerable upper intake level (UL) is 400 μg/day selenium, considering selenosis as the adverse effect [37]. This is generally related to the well-established pro-oxidant effects observed when using supra-nutritional doses of selenium. However, such pro-oxidant properties can also be potentially exploited for the development of novel therapeutic tools (vide infra).

While considering all these issues, there is a general agreement about the multiple positive roles of selenium on human health, and a general U-shaped non-linear relationship between selenium status and beneficial effects has been suggested. Overall, subjects with low selenium levels at baseline could benefit from supplementation; on the contrary those with an adequate or high status might be negatively affected [38]. In this scenario, the study of small molecules that contain selenium has also received considerable attention. Indeed, a broad range of organoselenium compounds have been demonstrated to possess remarkable biological properties. For example, the benzoselenazole derivative ebselen—arguably the most studied selenium-containing small molecule—exhibited a wide array of valuable biological functions. Selenium-containing synthetic small molecules proved their potential role also as anticancer and antibiotics [39]. Ebselen is an inhibitor of TrxRs, particularly in prokaryotic cells, that plays a central role in counteracting ROS, thus leading to an intracellular elevation of reactive oxygen species (ROS). A number of selenium-containing molecules also exhibited remarkable carbonic anhydrase inhibitor activity against different pathogenic bacteria, including *Vibrio cholera*, *Burkholderia pseudomallei*, and *Mycobacterium tuberculosis* [40]. Over the past years, the synthesis and the study of synthetic or semi-synthetic selenium-containing derivatives has attracted a steadily growing interest amongst organic chemists, medicinal chemists, and biologists [41,42,43,44,45,46,47,48,49].

Here we report the main literature results about the relationship between selenium and some of the most important present-days diseases, considering the milestones of research about these topics, with particular emphasis on the most recent results.

## 2. Cancer

A great interest about cancer chemoprevention by selenium dates back to the late 1960s. In 1966 Shamberger and Rudolph showed that sodium selenide (Na_2_Se) greatly reduced tumour formation in an induced mouse skin tumour model [50]. The anticancer role of selenium was also empirically speculated on the basis of the inverse relationship existing between cancer mortality rates and selenium contents in blood and forage crop in the United States [51,52].

Since 1970, numerous epidemiological selenium supplementation studies and clinical trials supported the “Selenium−cancer hypothesis” linking a low selenium intake with a higher incidence of cancer. The 1996 study led by Clark and Combs on a population of 1312 patients was considered the zenith of this research field, showing that supplementation with 200 μg/day of selenium in the form of selenized yeast significantly reduced colon, prostate, and lung cancers in a multicentre, double-blind, randomized, placebo-controlled cancer prevention trial. The selenium supplementation was also showed to significantly reduce total cancer mortality over a 10-year time period [53].

Since the first studies, cancer patients showed lower pre-diagnostic serum selenium levels than controls; selenium treatment reduced tumour yield in animal models [54].

**Figure 2:**
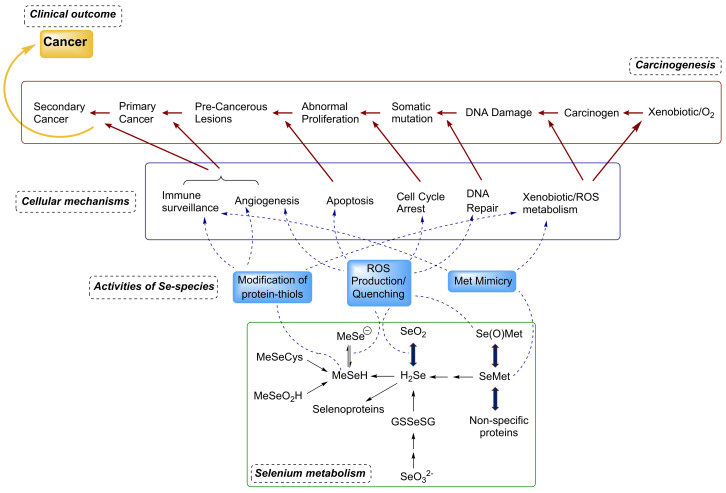
The multiple-stage action of selenium on cancer-related pathways [56].

Selenium exerts its chemo-preventive effect primarily by maintaining the correct redox homeostasis and an error-free protein folding, mainly through selenoproteins such as glutathione peroxidases (GPxs), thioredoxin reductases (TrxRs) and selenoprotein P (SelP, SeP or SELENOP), that prevents DNA oxidative mutagenic stress [55]. Other functions include the modulation of gene expression, the redox and hormonal regulation of metabolism, and a role in DNA repairing and cell-signalling pathways. Selenoproteins act at different pivotal levels: they inhibit cell proliferation, stimulate apoptosis, and reduce metastasis arresting the cell cycle in the G1 phase, via the redox modification of protein-thiols, and methionine mimicry in critical proteins (Figure 2) [56,57,58]. Selenoproteins that are directly or indirectly linked to redox homeostasis maintenance, such as GPXs, TXNRD1, SELENOF, and SELENOP appear to affect multiple signalling pathways involved in cancer initiation and progression. A decreased selenium status may imbalance these pathways by affecting the abovementioned selenoproteins synthesis, thus resulting in tumour initiation and progression, as in the case of colorectal cancer (CRC). Furthermore, there is strong evidence that single nucleotides polymorphisms affecting some selenoproteins (GPX-1, GPX-2, SELENOP, TXNRD1) may support the development or progression of CRC [59].

SELENOH is a key regulator for cell cycle progression and its expression is highly dependent on selenium status. SELENOH KO in vitro and in vivo (on human colorectal cancer cells) unexpectedly increased proliferation and migration, highlighting a role of SELENOH in inhibiting tumour progression and in protecting colorectal cancer cells from uncontrolled proliferation [60].

TrxR2 over-expression in cancer cells has often been pointed out as a key factor for tumour development and progression, as well as for apoptosis resistance. Inhibition of TrxR2 causes an increased mitochondrial concentration of reactive oxygen species, via the impairments of the Trx2 activity, which results in the release of a number of pro-apoptotic factors such as cyclophilin D. Thus, a selective inhibition of TrxR2 could be utilized as a strategy to kill cancer cells inducing apoptosis [61].

Beyond selenoproteins, different metabolites act at different stages for tumour prevention. Methylselenol, generated in the body from inorganic or organic selenium compounds, is arguably one of the most important among such metabolites [55,56]. Active selenium metabolites include: (i) selenodiglutathione (GS-Se-SG), the reductive metabolite of oxidized inorganic salts (selenite, selenate); (ii) selenomethionine (SeMet), a selenated methionine analogue which represents the main form of selenium in food; (iii) hydrogen selenide (H_2_Se), the common intermediate of the reductive pathway and the catabolism of seleno-amino acids; (iv) methylated metabolites of selenide such as CH_3_SeH (methylselenol), (CH_3_)_3_Se^+^ (trimethylselenonium), CH_3_SeCys (methylseleno-cyeteine) and CH_3_SeO_2_H (methylseleninic acid). Such metabolites play various roles that influence the selenium anticarcinogenesis both at underlying and intermediate levels (Figure 2). For example, GSSeSG has been reported to enhance apoptosis, inhibit the DNA-binding of AP-1 (activator protein 1) transcription factor, and inhibit cell proliferation. Methylated selenium derivatives induce apoptosis in breast carcinoma, hepatoma and neuroblastoma cells [56]. Their possible role in inhibiting neo-angiogenesis of activated endothelial cells has also been reported. Indeed, precursors of methylselenol have been shown to inhibit the expression of vascular endothelial matrix metalloproteinase-2 and growth factor in cancer cells [56,58].

At high supra-nutritional doses, different mechanisms over the maintenance of the redox homeostasis take place. For example, selenolates can be involved in killing cancer cells by the production of superoxide, that drives the cancer cell towards an irrecoverable oxidative status and then to the apoptosis. Selenite and methaneseleninic acid, common forms of selenium in biology, can both oxidize thiol groups of enzymes, leading cancer cells toward apoptosis [62]. Typically, selenium compounds exert their cytotoxic effects by acting as pro-oxidants and, therefore, altering the tumour cellular redox homeostasis, also preventing metastasis formation with significant specificity and efficiency, alongside with reduced side effects. In this context, a number of novel organoselenium compounds have been recently synthesized and tested as potential chemotherapeutic agents [63].

Generally, such compounds act by inducing apoptosis in cancer cells, mediated by caspases, mitochondrial dysfunction/signalling, ER stress, ROS production and oxidative damage, DNA degradation/fragmentation, or cytoskeleton damage. In some cases, the induction of autophagy has been shown to be caused by the specific inhibition of multiple kinases. The observed mechanisms depend on the cancer type and the different nature of the molecule [64]. For example, in certain prostate cancer cell lines, methylseleninic acid (MSA) has been demonstrated to selectively react with some thiol moieties close to the catalytic domain of protein kinase C (PKC) antiapoptotic isoenzymes (ε and α), inactivating them and leading cells to apoptosis. This is a biphasic effect as lower concentrations of MSA-induced cell death, while higher concentrations deactivate proapoptotic PKC isoenzymes (δ and ζ) and caspase-3, rendering tumour cells resistant to apoptosis [65].

Notably, genetics, gender, and modifiable behaviours modulate the impact of selenoproteins allelic variants on carcinogenesis. Particularly, the interaction between genetic factors and the dietary selenium intake seems to be effective in determining cancer risk and outcome via the metabolism of pivotal selenoproteins such as SelP, SelF (Selenoprotein F), GPx4, and GPx1 [66]. Some polymorphisms may be associated with the increase of aggressive prostate cancer, breast cancer and colorectal cancer [55].

Thus, selenium-deficient individuals and those with allelic variants of certain selenoproteins show an increased cancer risk. On the other hand, while GPx2 seems to act preventively at the very early stages of cancer or when carcinogenesis is driven by an inflammatory state, when the cancer cell is already initiated GPx2 seems to support cell proliferation and tumour growth, also enabling a better survival for metastatic floating cells [67].

Several randomized controlled trials have been conducted over years in humans to determine the efficacy of selenium in reducing cancer risk, showing conflicting results [54,68]. In the first studies the administration of selenium-enriched table salt proved to be effective against primary liver cancer [69,70] and selenium-containing multiagent supplements were effective against oesophageal cancer [71,72], precancerous oral lesions [73,74], non-melanoma skin cancer [53,75] and prostate cancer [76,77].

Later research showed contrasting evidence, as reported by Rayman [2] and other authors [1] who provided a comprehensive discussion of the main results on this topic up to 2012 and 2014 respectively. More recent studies highlighted that low selenium concentration in plasma was associated with a 4- to 5-fold increased risk of prostate cancer, in a case-control study that included 318 patients [78]. However, this result is in contrast both with evidence from the previous Selenium and vitamin E Cancer prevention Trial (SELECT) study [75,79,80] and with the results of a 2020 review of randomized controlled trials [81]. Some authors have suggested that divergent results could be primary due to the different specific cancer type considered, as well as to the selenium form and to the initial plasma selenium levels of the participants in the trials [82]. A 2018 review of 37 studies conducted in different geographical areas on different cancer types consistently confirmed that selenium supplementation may be protective against cancer but with different effects according to the specific tumour [82]. More recently, 481 men were supplemented with 200 µg/d selenium in the form of selenized yeast for six months. Selenium and prostate-specific antigen (PSA) levels were measured in serum at pre-and post-supplementation. Overall, there was no significant correlation between changes in PSA and changes in selenium levels by supplementation, showing the interactive influence of supplemented selenium with demographic, lifestyle, genetic and dietary factors, on prostate stability measured through serum PSA. This highlights the importance of optimizing serum selenium levels on a personalized scale, rather than depending on a continuous single dose selenium supplement, for prostate health benefits [83].

Evans et al. evaluated the possibility to assess the clinical potencies of the main nutritionally relevant forms of selenium and the relationship between their pharmacokinetic (PK) profiles and pharmacodynamics (PD) effects in cancer patients. Sodium selenite (SS), Se-methylselenocysteine (MSC) and seleno-L-methionine (SLM) were compared in two cohorts of 12 patients, one cohort with chronic lymphocytic leukaemia (CLL) and the other with solid malignancies [84]. In a previous clinical trial, all three Se compounds were demonstrated to be well-tolerated and non-genotoxic [85]. The 24 patients were orally administrated with 400 μg of selenium at random in the form of SS, MSC or SLM for eight weeks. No substantial changes were noted and according to the authors the dose examined in this cohort was too low to achieve an effective Se plasma concentration and thus to elicit significant PD effects. On the basis of pre-clinical data, the authors hypothesize that a dose escalation to supra-nutritional plasma levels may be required to generate meaningful changes in pharmacokinetic markers; this may confer therapeutic synergy against malignant cells and cytoprotection of healthy tissues when selenium compounds are administered concomitantly with anticancer therapies. Next studies on a subsequent cohort at higher doses are planned to evaluate PK and PD at higher supra-nutritional doses of selenium with the aim of obtaining a greater effect on patients and a better insight in the PK–PD relationship of each Se compound for cancer therapy [84].

Selenium optimal doses and physiological status correspond to an antioxidant activity that can be exploited for cancer prevention. On the other hand, the prooxidant properties of selenium can also be exploited to develop potential therapeutic tools for cancer treatment. Among a variety of organic and inorganic forms of selenium, Se-containing nanoparticles have attracted considerable interest owing to their interesting properties (i.e., reduced toxicity and improved targeting with respect to other Se-containing species). Several studies focusing on potential applications of Se-nanoparticles in cancer, ranging from chemotherapy, to diagnosis and anti-cancer drug delivery, have been reported [86].

Vinceti et al. recently concluded that the exposure to supra-nutritional levels of organic selenium could be related to an increased cancer risk [87]. Vernia et al. [88] highlighted the potential benefit deriving from the dietary intake of some microelements, including Se, on colorectal cancer. On the other hand, supra-nutritional selenium supplementation has been associated with detrimental effects on colorectal cancer (CRC). Selenium-repleted subjects, that are involved in the majority of supplementation clinical trials, may not benefit from selenium supplementation as would patients with nutritional selenium deficiencies or with SELENOP SNPs [89].

A very recent meta-analysis on 18 case-control studies investigated the relationship between selenium levels in human tissue and breast cancer risk, highlighting a negative correlation [90]. In this context, a similar study [91] focusing on breast cancer in obese patients, showed that decreased levels of selenoproteins in the adipose tissue of obese subjects resulted in an inflammatory state that may progress to cancer.

With reference to thyroid cancer, according to a very recent study [92] the link between selenium and thyroid cancer is inconclusive, because it is still unclear whether low selenium levels are a predisposing factor or a consequence. This is consistent with the results of previous works [93,94] that had already highlighted a poorly significant or no significant effect of selenium supplementation in thyroid cancer and thus the impossibility to establish a cause–effect relationship.

Very recently some experimental studies conducted using HeLa cells (a human cervical carcinoma cell line which contains HPV18 DNA) and mouse models of cervical cancer, either induced by HeLa cell transplantation, MCA (3-methylcholanthrene) exposition or human papillomavirus (HPV) exposition were also reported. Notably, HPV exposition-induced cellular cervical cancer model seems to be the most reliable mimic of the in vivo carcinogenesis process. The results highlighted an anticancer role of selenium nanoparticles (Se-NPs) against HPV and chemical carcinogen agents. Se-NPs enhance the targeting of specific drugs against cancer cells, increasing their effectivity at low doses. Most importantly, Se-NPs were shown to be non-toxic to non-cancer cells [95]. In fact, pivotal differences exist in the oxidative metabolism between tumours and normal tissues. This may represent the target for novel small therapeutic molecules, including selenium-containing derivatives, that simultaneously behave as pro-oxidant in neoplastic cells and antioxidant in healthy cells [58]. Indeed, the possibility to employ selenium or selenium-containing molecules for cancer treatment has recently emerged. In this context, and differently from the use of selenium in the prevention of cancer—which relies on its antioxidant properties—the therapeutic action of selenium is mainly centred around its pro-oxidant activity. For example, in a cohort of 45 patients with gynaecologic cancers including epithelial ovarian cancer, cancer of the fallopian tubes and cancer of the peritoneum, selenium was administered in the form of selenious acid in addition to the carboplatin/paclitaxel chemotherapy. A dose of up to 5000 μg resulted safe and well tolerated and it was suggested it might have a synergic interaction with cytotoxic drugs normally uses as chemotherapeutic agents [96].

An adequate selenium intake is required for the biosynthesis of antioxidant proteins, including glutathione peroxidase and thioredoxin reductase. In case of selenium deficiency, supplementation can improve the expression of selenium-dependent proteins and enzymes involved in the regulation of cellular redox status. On the other hand, a number of studies highlighted that the use of supra-nutritional levels of selenium is associated with positive effects without significant toxicity. Under such conditions, the expression of antioxidant proteins is not enhanced with respect to levels of subjects administered with the RDA, since those enzymes become saturated in the body at the suggested dietary intake of selenium. Therefore, the modulation of the selenium-related antioxidant system cannot account for the potential beneficial properties of high-dose selenium in cancer treatment. In this regard, the pro-oxidant activity of selenium is thought to play a crucial role. Indeed, depending on the cellular oxidative stress status, redox-active selenium compounds have been proposed to catalyse the oxidation of key proteins and to promote DNA damage, thus triggering apoptosis [97]. For example, the selenium-catalysed oxidation of thiol moieties of proteins and cysteine residues of glutathione (GSH) leads to intramolecular disulphide bonds. Thiol functionalities also react with selenium to form selenotrisulfides (dithiaselanes or selenium(II) dithiolates, RSSeSR) and selenenyl sulphides (RSSeR) [98,99]. When involving cysteine residues of catalytic domain of enzymes, these reactions can inactivate signalling molecules; for example, selenium-promoted oxidation of cysteine residues of NF-κB and AP-1 reduces the binding affinity of such transcription factors for target DNA [98,100]. Similarly, thiol functionalities of a number of proteins including, amongst others, redox-dependent signalling molecules such as caspase-3, Cyclin Dependent Kinase 2 (CdK2), protein kinase C, and c-Jun N-terminal kinase (JNK) have also been shown to undergo selenium-mediated oxidation, which potentially disrupts signal transduction pathways controlling cell survival and apoptosis [98,101,102,103,104].

Transformed cells are characterised by high oxidative distress, with consequently elevated levels of superoxide anion and hydrogen peroxide. Under these conditions, the pro-oxidant properties of selenium might offer an attractive opportunity to develop new therapeutic tools for cancer treatment. Furthermore, selenium-containing compounds such as selenite, selenium dioxide, and diselenides, are involved in the endogenous generation of superoxide anion, readily converted to hydrogen peroxide (H_2_O_2_) by superoxide dismutase (SOD). Highly reactive hydroxyl radicals (HO^•^) and nitrogen dioxide radicals (NO_2_^•^) can be formed from hydrogen peroxide in the presence of Fe^2+^ via Fenton reaction and upon reaction of hydroxyl radical with nitric oxide (NO^•^), respectively [98,105]. Thus, selenium exerts its pro-oxidant activity at three different levels: (i) selenium-catalysed oxidation thiol moieties of proteins and enzymes; (ii) reaction of selenium with glutathione (GSH) leading to its depletion; (iii) production of superoxide anions and generation of highly reactive species such as H_2_O_2_, HO^•^, NO_2_^•^.

Optimal dietary levels of selenium ensure the maintenance of the selenium-dependent redox homeostasis—also via a proper synthesis of antioxidant selenoproteins—which seems to be involved in preventing the onset of a number of diseases including—amongst others—cancer, cardiovascular disorders, neurodegenerative diseases and fertility impairments. On the other hand, although over-supply of selenium causes toxicity and may increase the risk of endocrine system disruption, mental disorders and cancer [106], supra-nutritional doses of selenium-containing compounds can be employed as chemotherapeutic agents for their pro-oxidant and pro-apoptotic action against cancer cells [86,107].

Therefore, the role of selenium and selenium-containing compounds in cancer could be both preventative and therapeutic. For example, COS-Se, a non-toxic conjugated molecule of chitosan oligosaccharide (COS) and selenium recently displayed great potential as a functional food ingredient in cancer prevention. COS-Se inhibited proliferation and metastasis of human gastric cancer cells SGC-7901 with non-toxic effects on the normal fibroblast L-929 in vitro. A supplementation with this molecule could significantly repress the growth of gastric adenocarcinoma by reducing levels of CD34 protein, vascular endothelial growth factor, and matrix metalloproteinase-9. Moreover, a COS-Se treatment could effectively elevate phagocytosis and increase the secretion of anti-inflammatory cytokines. Further experiments have shown that COS-Se exhibited immune-enhancing effects in mice models [108].

Novel seleno-derivatives drugs may also be used as alternative potential therapeutic strategy against glioblastoma, the most aggressive primary brain cancer in adults [109]. Additional studies are needed in order to understand the interplay of all the processes described above with individual metabolic differences and to confirm the relationship between selenium concentrations and cancer risk, determining the benefits from increased selenium intake. In particular, different tumour stages and the patients characteristics such as sex, age and selenium at baseline need to be taken into account [67].

## 3. Inflammatory States

Optimal levels of selenoproteins may be clinically beneficial in inflammatory disorders, especially when they have high peroxidase activity. In fact, oxidative stress from reactive oxygen and nitrogen species (ROS and RNS) results in different diseases where inflammation underlies. Inflammation impairs healthy cells that thus evolve towards degeneration, with the onset of several clinical condition including cancers, atherosclerosis, diabetes, autoimmune disorder, rheumatoid arthritis, pancreatitis, asthma, and neurodegenerative disorders.

**Figure 3:**
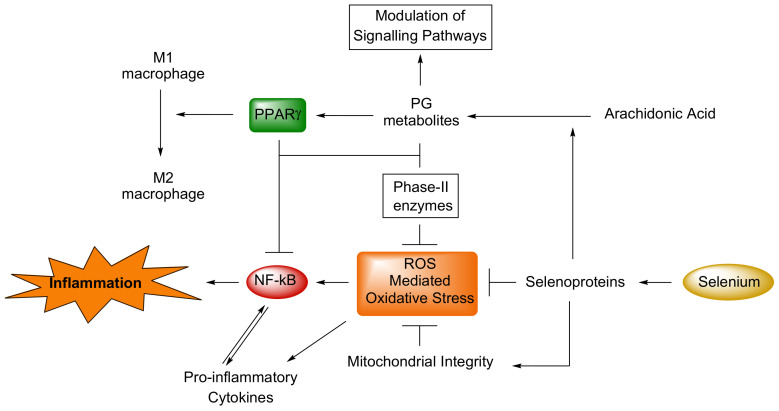
Selenium action on metabolic pathways of inflammation [110].

The multiple mechanisms underlying the anti-inflammatory role of selenium and their interrelations have been comprehensively discussed by Kaushal et al. [110]. Literature have reported data focused on the potential role of selenoproteins against ROS and on the relationship between cellular redox state and the activation of cyclo-oxygenases (COX) and lipoxygenases (LOX). These enzymes are involved in the production of lipid mediators such as prostaglandins (PGs), thromboxanes (TXs), prostacyclins (PGI2), leukotrienes (LT) and oxidized fatty acids, that are well-known biomarkers of inflammation released from tissues and immune cells in response to stress, free radicals, and infections. Such molecules are also involved in the fine modulation of pivotal metabolic signalling pathways as well as in the conversion of pro-inflammatory macrophages M1 to anti-inflammatory macrophages M2 (Figure 3).

Selenium deficiency may cause a reduced GPx activity that indirectly regulates the expression of COX and LOX via the Mitogen-activated protein kinase (MAPK) pathway and Cyclooxygenases-2 (COX-2), by controlling the Nuclear Factor kappa-light-chain-enhancer of activated B cells (NF-κB), the “central mediator of immune and inflammatory responses”. A number of naturally occurring dietary supplements and nutrients, including selenium, may modulate low-grade inflammation [110] and support anti-inflammatory mechanisms by suppressing such mediators.

High plasma levels of C-reactive protein (CRP), another common biomarker of inflammation, are associated with reduced serum selenium levels [111]. Low selenium levels may further trigger an increased ROS and RNS production up to Systemic Inflammatory Response Syndrome (SIRS) and sepsis, with extensive tissue damage and organ failure [110,112]. Selenium supplementation has reduced mortality under these conditions [113]. Redox imbalance is indeed closely related to the occurrence and development of a number of diseases. In this context, antioxidant-based therapies can be considered as an attractive option. On the other hand, cellular signalling pathways are strictly dependent on a physiological ROS level. Thus, precise redox strategies are necessary and redox status should be considered in the context of species, time, place, level, and target, to set appropriate trials and individualized therapeutic strategies or nutritional supplementation protocols [114].

**Figure 4:**
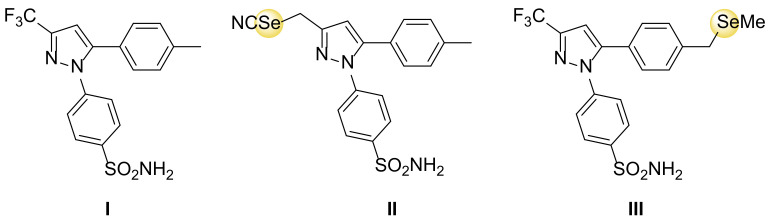
Structures of celecoxib (**I**), selenocoxib-2 (**II**), and selencoxib-3 (**III**).

Selenium-derivatives of Celecoxib (Figure 4), a well-known non-steroidal anti-inflammatory drug that selectively inhibits COX-2 activity, have been developed and tested in clinical trials for the prevention of colon cancer [115]. These novel molecules act on inflammatory processes that are preliminary to carcinogenesis thus conjugating anti-inflammatory and chemo-preventive effects. Notably, such molecules can be used at extremely low doses, limiting the typical side effects [116].

Recent epidemiological studies have reported that a selenium deficiency in patients with inflammatory bowel diseases (IBDs) [38,117] was related to an increased severity of Crohn’s disease and ulcerative colitis. Although IBDs have a multifactorial pathogenesis and different symptoms, they share a common chronic inflammatory condition of the intestine. The high expression of the four GPx isoforms in the enterocytes of IBDs patients suggests a compensatory mechanism to reduce the high levels of free radicals produced by immune cells during the inflammatory process, mediated by the transcription factor Nrf2. If adequate levels of selenium are available, Nrf2 can enter the nucleus and bind to antioxidant/electrophile responsive element (ARE/EpRE) regions to enhance the expression of antioxidant genes, including GPxs and TrxRs. These selenoproteins combat oxidative stress, attenuate inflammatory signalling pathways, and increase the population of anti-inflammatory M2 macrophages, helping patients to extend the remission phase [117]. Dietary selenium and selenoproteins have been observed to modulate specific pathways associated with such diseases. More specifically, aselenium and SELENOP deficiency will activate the WNT pathway (that has a role in carcinogenesis) and modulate production of inflammatory cytokines. The exact mechanisms by which this occurs should be further investigated and clarified [89].

The possible role of GPx in modulating the effect of inflammation in rheumatoid arthritis patients has been very recently investigated in a review of clinical and preclinical studies. In such a study the authors concluded that it is not clear whether selenium deficiency is a cause or a consequence of autoimmune inflammatory diseases [118]. In a very recent study performed on a Chinese cohort selected from an area with average supra-nutritional selenium intake, blood samples were taken from a group of patients with rheumatoid arthritis and from a second group of healthy controls. Generally, patients with higher selenium levels showed milder symptoms and lower levels of C-reactive protein, IL-6 and RANKL. After further test in vitro the authors concluded that a high selenium intake might regulate RANKL expression via ROS modulation [119]. Overall, the maintenance of an adequate redox homeostasis, also through the intake of these nutrients, seems to be helpful to prevent or relieve the effects of inflammatory diseases. An adequate selenium intake might ensure the correct functioning of antioxidant pathways, including those involving selenoenzymes.

## 4. Cardiovascular Disease

Oxidative stress impairs endothelial cell function contributing to the onset and progression of some cardiovascular diseases (CVD). Cyclo-oxygenases (COXs) are the major enzymes producing five different prostanoids that have vasoconstricting or vasodilating activity. Under conditions of increased oxidative stress, the altered expressions and activities of COXs affect the vascular tone with increased risk of cardiovascular manifestations. Novel drugs targeting oxidative stress, COX-2 and prostanoids against common cardiovascular diseases have been evaluated in recent years. Experiments on animal models have demonstrated that selenium decreases oxidative stress and COX activity, downregulating the leukotriene pathway in diabetic cardiac hypertrophy [120].

Nitric oxide (NO) is the most potent endogenous vasodilator. It also inhibits smooth muscle cell proliferation and migration, adhesion of leukocytes to the endothelium, and platelet aggregation, thus its correct turnover is crucial for endothelial function [121]. Vascular homeostasis is maintained by an equilibrium between the generation of reactive oxygen species (ROS) and NO production [122] and it depends on the constitutive endothelial nitric oxide synthase (eNOS) and on the vascular NAD(P)H oxidases. This delicate balance is influenced by the superoxide anion/nitric balance that has been shown to be modulated by selenium via the action of some selenoproteins like cGPx and TrxRs. These act at different levels, such as the scavenging of reactive oxygen species (that would imbalance the NO/superoxide ion ratio), and the prevention of oxidative inactivation of pivotal enzymes (such as eNOS and vascular oxidases). For example, a TrxRs overexpression has prevented the inactivation of eNOS in genetically modified porcine pulmonary artery endothelial cells, while a cGPx deficiency induced by genetic alteration caused the depletion of bioavailable NO through the inhibition of NOS [121].

It has been recently observed that the regulatory protein caveolin-1 inactivates eNOS by allosterically competing with the calcium-dependent activation of eNOS by calmodulin. This reduces nitric oxide bioavailability by reducing NO production, leading to endothelial dysfunction. High expression levels of caveolin-1 were associated with the administration of high concentrations of selenite. Interestingly, a physiological concentration of selenite decreases the mRNA expression of caveolin-1 [122].

Different evidence has emerged in cells equipped with inducible nitric oxide synthase (iNOS), such as macrophages [121]. In selenium-deficient RAW 264.7 (a line of mouse murine macrophage cell), an enhanced expression of iNOS has been observed, mediated by the upregulation of the redox-sensitive transcription factor NF-κB. Consequently, an increased production of NO was registered, with an inflammatory associated condition due to increased levels of oxidative stress [123]. Similarly in pig brains, a selenium deficiency activated the iNOS/NF-κB pathway, upregulating the expression of inflammatory cytokines and leading to inflammatory lesions [124]. Selenium-containing supplements proved to be effective in blocking cytokine-induced upregulation of NF-κB and iNOS, thus reducing stress-related NO inflammatory levels in the hypothalamus and hippocampus of an animal model [125]. Selenium feed supplements in chickens alleviated cardiac injury, splenic lymphocytes, and splenic tissue damage associated with Cd-derived or Hg-derived inflammation, via the production of NO through the NF-κB/iNOS pathway [126,127].

A mechanism has been proposed for the regulatory action of selenium on NO synthesis that involves the inhibitory effect of GPx on the expression of iNOS, both deriving from inorganic or organic selenium [123,128].

Thus, an adequate intake of selenium may exert a potential preventive effect against non-infectious CVD. A synthetic overview of recent studies focusing on the role of selenium in cardiovascular diseases is reported in Table S1 (see Supplementary Materials). Selenium has already been recommended as a therapeutic measure in cardiovascular diseases to block IL-15-dependent epithelial damage and inflammation-linked complications [110]. However, several epidemiological studies based on observational data and clinical trials aimed to clarify the relationship between selenium and cardiovascular health, without any conclusive response [1]. A general agreement ascribes the inconclusive results to differences in baseline selenium levels, to the influence of other co-supplemented antioxidants and to the different protocols (supplementation strategies and selenium species used) [129,130,131,132,133].

**Figure 5:**
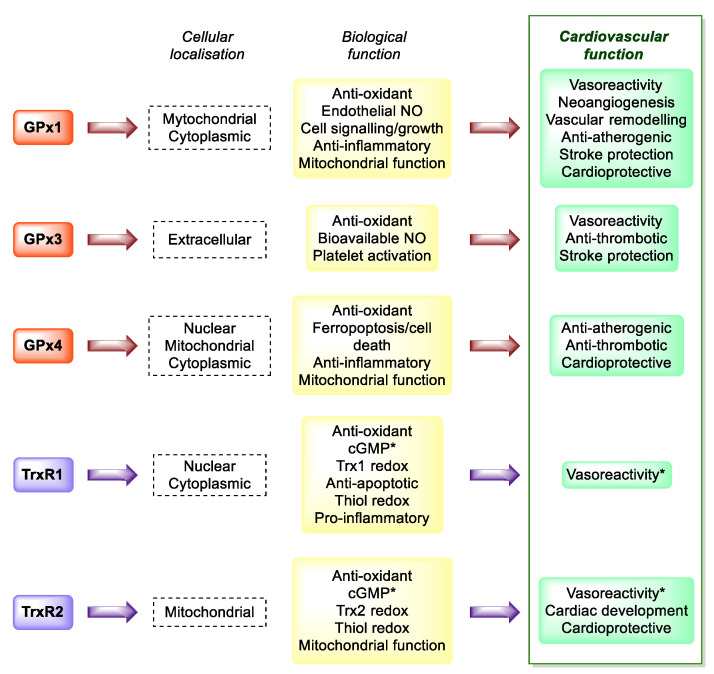
GPxs and TrxRs and their relationships with cardiovascular health. * It is unclear whether both TrxR1 and TRxR2 contributes to this function [134].

Experiments focusing on the role of selenium deficiency for cardiovascular diseases (without infectious origin) have shown that the association between low selenium intake and cardiovascular pathologies might result from an increased oxidative stress and its consequences [129]. As early as 80 years ago a low Se intake has been associated with a rapidly progressive cardiomyopathy, resulting in extensive fibrosis and degenerative changes, today named “Keshan disease”. The onset of this myocarditis is known to be caused by a coxsackie virus, whose virulence is increased in condition of selenium deficiency and reduced GPx1protective activity [2]. Indeed, the redox active selenoproteins GPxs and TrxRs protect cardiovascular system by preventing or modulating the oxidative stress and the oxidative modification of lipids, reducing inflammation, limiting platelets aggregation, and maintaining the correct vasoreactivity, that are all main risk factors for coronary and heart failure [2,134] (Figure 5).

Selenium supplementation has proven to be effective in reducing oxidative damage after cardiac ischemia-reperfusion, via an increased GPxs and TrxRs function [135]. Selenium supplements might thus help to maintain the general redox homeostasis and to reduce the risk of cardiovascular disease and associated mortality, as suggested by studies in human subjects. Further investigations are needed in order to better clarify the specific mechanisms of action of the involved selenoproteins.

Selenoprotein TrxR1 is needed to reduce coenzyme Q10 (ubiquinone, CoQ_10_) to its active form ubiquinol and a selenium deficiency could impair the cell from obtaining optimal concentrations of CoQ_10_. Coenzyme Q_10_ is a central electron carrier in the mitochondrial respiratory chain and is also a powerful antioxidant, mainly acting against lipid peroxidation. Patients with cardiomyopathy and those with ischemic heart disease showed low concentrations of CoQ_10_ and its supplementation increased the cardiac and endothelial function, as shown by different meta-analysis. Furthermore, CoQ_10_ levels were revealed to be positive prognostic indicators of risk of cardiovascular death [136]. A prospective double-blind, placebo-controlled study was carried out in a population of 443 elderly participants that were administrated a supplement of selenium and coenzyme CoQ_10_ in a four-year long intervention. The results of a 10-year follow-up showed reduced cardiovascular mortality, a better cardiac function and a lower plasma concentration of the biomarker NT-proBNP [136,137]. Intriguingly, patients with lower selenium concentration showed the highest mortality. The cardio-protective effects of selenium supplementation were observed in subjects with low selenium concentration, while those with no selenium deficiency were not affected by selenium supplementation [138].

An explanation for the positive clinical effects previously reported was later proposed on the basis of a lower fibrosis condition [139] and/or in an increased concentration of insulin-like growth factor-1(IGF-1) and insulin-like growth factor-1 binding protein (IGFBP-1) in supplemented subjects compared with placebo. In this context, IGF-1 has been reported to have significant anti-inflammatory and antioxidant effects [140]. The protective action of selenium supplementation persisted in the follow-up period and was still observed after 12 years [141]. Additionally, a very recent sub-study on a sub-group of 219 subjects from the same original population highlighted a significant decrease in fructosamine concentration as a result of the supplement intervention, especially in patients with lower selenium levels at baseline. Notably, fructosamine is a long-term marker of glycaemic control in diabetic patients and it is also a marker of cardiovascular risk [142].

In a 48-month long randomized, double-blind, placebo-controlled trial 213 subjects were administrated with selenium yeast (200 µg/day) and CoQ_10_ (200 mg/day) or placebo. At baseline and at the end of the trial D-dimer was measured. D-dimer is a product of fibrinolysis that is used as a biomarker of endothelial dysfunction, thromboembolism, and inflammation, and is associated with cardiovascular mortality in ischemic events. All the subjects in the cohort presented low selenium levels at baseline (mean 67 µg/L, SD 16.8). The individuals with a D-dimer level above median at baseline, showed a significant benefit from supplementation, resulting in lower cardiovascular mortality compared with the placebo group [143].

The combined supplementation of selenium and CoQ_10_ thus seemed to reduce cardiovascular mortality by preventing the D-dimer increase. Thus, although results of the abovementioned studies suggest a synergic action of selenium and CoQ_10_ against cardiovascular disorders, selenium supplementation may be considered as a rewarding intervention strategy only in patients with low selenium levels at baseline.

Iodothyronine deiodinase 1 (DIO1) also plays a role in cardiovascular health: a change in circulating lipoproteins occurs in case of hypothyroidism, with an increase of the atherogenic features. An adequate activity of DIO1 seems to be important for the homeostasis of lipid metabolism [1]. A meta-analysis of 25 observational studies that measured blood or toenail selenium concentrations (14 prospective cohorts, 11 case-control studies) and 6 randomized trials of selenium supplementation [144] had already found a statistically significant, moderate inverse correlation between total selenium concentration and coronary heart disease (CHD) risk. People with lower selenium concentrations were found to have a higher risk of CHD, especially in populations with low selenium intake. Increased selenium levels corresponded to a lower incidence of CHD, in particular in subjects with low dietary Se-intake. On the other hand, in subjects who already had an adequate Se-intake, overexposure could cause cardiovascular damage.

A 2017 review and meta-analysis of randomized controlled trials evaluated the effect of selenium supplementation on CHD mortality, blood lipid profile, serum C-reactive protein (CRP), and the level of GPx. This study considered 16 placebo-controlled and double-blinded trials for a total of 43,998 participants. Selenium supplementation decreased serum CRP and increased the GPx level, thus suggesting a positive effect on reducing the oxidative stress and inflammation that can exacerbate CHD. However, selenium supplementation was not enough to ameliorate the haematic lipid profile or reduce mortality [145].

With reference to the effects of selenium on haematic lipid profile, the mechanism underlying this connection remains partially unclear [146].

Although selenium may play a crucial role in lipid peroxidation and lipoprotein metabolism, this topic needs to be further investigated [133].

Early data from the French multicenter trial SU.VI.MAX showed no significant impact on ischemic cardiovascular disease incidence from selenium supplementation. Intriguingly, increased triglyceride and lowered HDL-cholesterol levels were found among men but not in women [146,147,148]. A post interventional study on the same cohort during a five-year follow-up showed that the total cholesterol and non–HDL cholesterol plasma levels were lower compared with the placebo group [149].

A randomized trial on the UK PRECISE cohort showed a significant reduction of total and non-HDL plasma cholesterol after a supplementation of low and medium doses of selenium. On the other hand, high supplementation increased the HDL-cholesterol, and the total/HDL cholesterol ratio decreased progressively with increasing selenium doses [150]. Similarly, 60 diabetic patients undergoing hemodialysis (HD) were randomized into two subgroups and administrated with 200 μg selenium per day or a starch placebo. After 24 weeks the supplemented patients showed significant reduction in serum insulin levels, insulin resistance, total cholesterol, LDL-cholesterol, and C-reactive protein with respect to the placebo group. Moreover, a significant increase was observed for the parameters of insulin sensitivity, HDL-cholesterol and total glutathione. Overall, the selenium supplementation improved the general metabolic status in HD diabetic patients [151].

The BIOSTAT-CHF observational study of 2516 subjects with heart failure showed a strong association between selenium deficiency (<70 μg/L in plasma) and mortality or hospitalization, reduced exercise tolerance and poorer quality of life [131].

On the other hand, a cross-sectional analysis on the random Kardiovize urban cohort, including 894 subjects, found no significant association between selenium intake (considered within a composite dietary antioxidant index) and Carotid intima-media thickness (cIMT). Selenium levels negatively correlated with other cardiovascular risk factors such as waist-to-hip ratio (WHR), body fat mass, (BFM), and total cholesterol/HDL ratio, and positively correlated with HDL-cholesterol. Intriguingly, such association was more significant in women. However, in this context it is worth mentioning that the specific contribution of the different dietary antioxidants to the overall results were difficult to assess [152].

In the NHANES study on 2903 participants, those with higher serum selenium levels showed lower rates of general and CVD mortality. The best protective effects were on subjects with a lower cardiovascular risk. Furthermore, while serum selenium was significantly associated with overall mortality in both genders, the relationship with CVD mortality was significant only among females [130].

Lower levels of serum selenium were found in 32 hospitalized patients with chronic heart failure (CHF) with respect to the healthy controls. Additionally, selenium levels showed a significant reverse relationship with left ventricular volume and pulmonary artery pressure [153].

A very recent case-control study investigated the possible association between plasma selenium levels and first stroke risk. A non-linear negative association between baseline plasma selenium levels and stroke risks was found in males but not in females [154]. Similarly, a very recent study investigated the relationship between trace elements, including selenium, and aortic valve sclerosis (AVSc), the thickening and calcification of the aortic valve described as the late outcome of a long-lasting inflammatory process. The patients group showed lower serum selenium concentrations compared with a healthy control group [155]. This appears to be consistent with the protection against ROS and RNS that some trace elements, including selenium, normally provide.

Despite the positive results reported above, several observational studies failed in finding a statistically significant relationship between selenium concentrations and risk of heart disease or cardiac death. Remarkably, associations between higher selenium concentrations and globally increased risk of CVD were also found [2,132,156]. In this context, a recent meta-analysis of 16 observational studies and 16 random control trials showed no significant effects of selenium supplementation on cardiovascular events on the considered cohorts. However, a possible inverse and U-shaped correlation between selenium levels and CVD risk was suggested [157]. These findings are in line with results that emerged from a comprehensive 2012 review [2]. The general conclusion of studies reported to date is that no further advantages derive by supplementing selenium beyond a certain plasma concentration. Therefore, these results generally do not support the use of selenium supplements for preventing heart disease, particularly in healthy people who already obtain sufficient selenium from food. Indeed, as mentioned above, an excess of selenium is capable of negatively influencing redox status via direct thiol oxidation and indirect generation of reactive oxygen species. Additional specific clinical trials are needed to better understand the contributions of selenium from food and dietary supplements to cardiovascular health, in particular for subjects that are Se-deficient.

## 5. Thyroid Disease

Selenium concentration is higher in the thyroid gland than in any other organ in the body. Together with iodine selenium plays an important role in thyroid hormone synthesis and metabolism. An inverse association between serum selenium concentrations and thyroid volume, risk of goitre, and risk of thyroid tissue damage was found in people with mild iodine deficiency. However, these results were statistically significant only in women.

A study identified a missense mutation of the SECIS-binding protein 2 (SBP2) gene to be responsible for some issues in thyroid function. These dysfunctions cannot be solved through selenium supplementation, and they may be due to the decreased activity of iodothyronine deiodinase 2 (DIO2) and to the lack of expression of iodothyronine deiodinase 1 (DIO1) and iodothyronine deiodinase 3 (DIO3) [158,159].

In other endocrine disorders, altered levels of iodothyronine deiodinase (DIO) may be due to an inadequate intake of selenium through diet: for example, the combination of insufficient selenium and iodine intake seems to be the cause of the endemic myxedematous cretinism [160]. Furthermore, a moderate selenium deficiency may be associated with autoimmune thyroiditis. Populations with an adequate iodine status but low selenium status are prone to increased prevalence of thyroid disease including Hashimoto’s autoimmune thyroiditis (AIT) [161]. Several interventional studies over the past few years have demonstrated a variable decrease of thyroid-peroxidase antibodies (TPOAb) in patients with AIT or Grave’s diseases supplemented with selenium in the form of selenomethionine, selenites or selenated yeasts [110,161]. As recently reviewed by Winther et al. other beneficial effects, such as reduced fatigue or reduced pro-inflammatory cytokines, were in some case achieved [161]. Similarly, Zuo et al. analysed the results of 17 trials reporting the apparently beneficial effects of selenium supplementation in patients with thyroid diseases, with decreased levels of FT3, FT4, and TPOAb [162]. Seventy-one children and adolescents with AIT were administrated with organic L-selenomethionine at the high dose of 200 μg or placebo daily for six months. Se supplementation appears to reduce anti-Tg in the Se group compared with the placebo group. No significant difference in thyroid gland volume was observed [163]. A cohort of 102 subjects aged 15–78 years was randomized into three groups treated respectively with 200 μg/day sodium selenite, 500 mg vitamin C/day or a placebo over a 3-month period. Thyroid stimulating hormone (TSH), TPO-Ab, antithyroglobulin antibody (Tg-Ab) and selenium concentrations were measured before treatment and at the end of the study. Notably, TPO-Ab concentrations decreased within the Se and vitamin C-treated groups, but not in the placebo group. These findings support the hypothesis of the beneficial, antioxidant effects of selenium in AIT even if it did not prove to be superior to vitamin C [164]. In a group including 47 women with Hashimoto’s thyroiditis and low vitamin D status, half were treated for 12 months with a 200 μg daily selenomethionine supplement and vitamin D (4000 IU daily). The second group received no selenium but only vitamin D supplement. The two study groups were well-matched for age, weight, titres of thyroid antibodies, levels of thyrotropin and free thyroid hormones. By analysing serum titres of thyroid peroxidase and thyroglobulin antibodies, as well as circulating levels of thyrotropin, free thyroid hormones, and 25-hydroxyvitamin D the authors concluded that the effect of exogenous vitamin D supplementation on thyroid autoimmunity was more pronounced in the supplemented group than in the native one, indicating possible benefits in a combined treatment of selenium and vitamin D for Hashimoto’s thyroiditis [165]. On the other hand, with regard to subclinical hypothyroidism, 42 patients were involved in a double-blinded clinical trial and randomized to daily receive 200 μg of selenium or a placebo. After eight weeks of treatment no significant effect on serum anti-TPO Ab and TSH levels was observed [166].

A common extrathyroidal manifestation of Graves’ disease (GD) is Graves’ orbitopathy, a consequence of the action of activated T lymphocytes not only against thyroid tissue but also against orbital tissue and extraocular muscles. A controlled, randomized trial was conducted at an ophthalmology referral centre in Mexico City. Patients were randomized into two groups and administrated with a placebo or a selenium supplement (200 µg/day) for six months. Pre-treatment values showed no statistically significant differences between groups. At the end of the treatment the supplemented group showed statistically significant differences in CAS score (which consists of seven inflammatory signs referred to the eyes) while no differences were found in any variables in the placebo group [167].

Hypothyroidism is a common occurrence during pregnancy or after childbirth which can have negative consequences for the mother and the new-born. In this context, different selenomethionine supplementation randomized control trials in pregnant women with hypothyroidism and subclinical hypothyroidism were compared [168]. Selenium supplementation proved to be effective in decreasing the incidence of moderate to advanced postpartum thyroiditis. Micronutrients deficiency is a common occurrence during pregnancy, that can impair correct foetal growth. A very recent prospective cohort study on 1931 pregnant women showed that maternal selenium status during pregnancy appears to be non-linearly associated with thyroid function and low thyroid function with low birth weight [169].

In this scenario, further research is needed to better understand whether selenium supplements can effectively support the prevention or treatment of thyroid disease [68,161] both in the general population and in pregnant women.

## 6. Fertility and Reproduction

Selenium deficiency causes impaired male fertility in livestock, laboratory animals, and humans. Since the beginning it has been clear that while a moderate selenium deficiency impairs sperm motility and morphology (up to the disconnections of head and tail), a severe deficiency completely precludes spermatogenesis [170,171,172]. Pioneeristic studies with radio-marked ^75^Se had already shown that selenium is accumulated in testis and epididymis into several proteins [173,174]. Recently, high resolution X-ray fluorescence microscopy (XFM) allowed a more sensitive characterization of selenium delivery and use in testis and sperm. Results of quantitative analysis on biological samples via inductively coupled plasma mass spectrometry (ICP-MS) or atomic absorption spectroscopy (AAS) also provided important information about selenium-containing species involved in male fertility [172].

The main testis selenoproteins is the Glutathione peroxidase 4 (GPx4), that occurs for about 50% in the keratin-like mitochondrial capsule of spermatozoa and is highly active in spermatids but inactivated in mature sperm [172,175]. Spermatozoa are particularly sensitive to high levels of reactive oxygen species (ROS) due to their limited antioxidant systems. High levels of ROS in spermatozoa cause lipids, proteins and DNA oxidation. Oxidative stress is associated with low sperm quality and male infertility. In several animal models, selenoproteins of the GPxs family proved to be efficient in protecting spermatozoa and its DNA from oxidative damage. Notably, this represents a key point for setting improved therapeutic strategies for men infertility [176]. The relation between redox homeostasis and the maintenance of male fertility has long been established. At physiological levels, ROS are essential for sperm function and fertilization, being involved in pivotal processes from steroidogenesis to the oocyte fertilization by spermatozoa. Under pathological conditions abnormal production of ROS may occur, impairing the male reproductive function. The inner antioxidant system mainly ensures the redox homodynamic maintenance; however, exogenous antioxidants obtained through the diet may have an important role in case the inner activity is not enough. On the other hand, unregulated supplementation can inhibit the above described processes that are fundamental for the reproductive function. Thus, the main challenge for assuring a correct male fertility is to maintain ROS at proper physiological concentration, by balancing oxidants and antioxidants [177].

GPx4 was found to reduce phospholipid hydroperoxides and H_2_O_2_ [1,178], that are involved in protamine sulfoxidation, fundamental for sperm concentration. On the other hand, phospholipid hydroperoxides and H_2_O_2_ also contribute to oxidative stress negatively affecting the structure and motility of spermatozoa. Thus, GPx4 reasonably performs an extremely fine modulator role for male fertility, protecting sperm cells from oxidative damage during maturation [172]. After reduction with GSH or other thiol reductants, GPx4 is restored in its active form. In addition to the mitochondrial form-mGPx4-, two other forms have been identified: the cytosolic form (cGPx4) [172] and the nucleus form (nGPx4) [179]. Initially it was unclear whether one of these forms in particular was responsible for the role of selenium in male reproduction or not. Specific studies [180,181] have suggested that only the mitochondrial isoform is important for male reproduction. A genetic study on 73 men demonstrated that GPx4 expression is decreased in about 10% of infertile men and about 35% of men with oligoasthenozoospermia, with significantly decreased sperm motility and spermatozoa concentration [182]. A different study [183] showed significantly lower GPx4 levels in sperm samples from 75 infertile men with respect to the controls. This was also correlated with spermatozoa viability, morphological integrity and forward motility.

A heterozygous mutation was identified in the SBP2 gene, leading to a lower expression of SBP2 in testis, with the arrest of spermatogenesis up to complete azoospermia [172]. Further investigations also demonstrated that the liver-secreted SelP—the only mammalian selenoprotein with more than one selenocysteine—is an indispensable source of selenium for testis [172] where, as expected, it plays an antioxidant role. Notably, transgenic SelP-null mice were affected by male infertility [184].

Another testis-specific selenoprotein is thioredoxin-Glutathione reductase (TGR or TRxR3) which was suggested to participate in disulphide bond isomerisation during sperm maturation, thus directly affecting male fertility [185].

Additionally, selenoprotein V (SelV) showed testis-specific expression in rodents, where it was found to be located especially in seminiferous tubules. Although its precise function in spermatogenesis still needs to be clarified, data about its structure, including a thioredoxin-like fold and a conserved CxxU motif, allow us to hypothesize a potential redox function. Very recently, SelV was shown to be protective against endoplasmic reticulum stress and oxidative injury induced by pro-oxidants. For these reasons it may be reasonable to hypothesize a protective antioxidant role on sperm [186].

A recent study by Salas-Huetos et al. [187] concluded that selenium supplementation in sub-fertile men with low selenium intake significantly increased the sperm quality parameters including sperm motility, semen volume, total sperm count and concentration, spermatozoa morphology, and increased the chance of conception. This might be the result of an improved function—at proper dietary intake levels of selenium—of the abovementioned selenoproteins.

Tellez Rojo et al. analysed the correlation between selenium intake and pubertal development in a population of 245 male subjects (from 10 to 18 years old). The study highlighted that a consumption of selenium below the RDA was associated with later pubertal development [188].

Nevertheless, different evidence has also emerged. In the Hawkes et al. intervention study 42 men were administrated for 48 weeks with a high selenium yeast supplement but no effects were observed on the sperm quality parameters neither in the positive nor in the negative [189]. The authors hinted that a self-regulation mechanism might protect testes from fluctuation of selenium levels. The MOXI multicenter, randomized clinical trial on 171 participants (including oligospermic and asthenospermic men), administrated a part of them with a multi-antioxidant formulation, including l-selenomethionine. Although the sperm concentration increased after the treatment with respect to the control group, no statistically significant differences were detected in the sperm morphology or motility or in the in vivo pregnancy rate. Although, according to this study, the combined antioxidant treatment did not improve semen parameters, the authors suggested that larger trials should be performed in order to better elucidate this topic [190]. Recently, Cannarella et al. suggested that selenium might be a possible non-hormonal therapeutic strategy for patients with chronic autoimmune thyroiditis—present in association with male infertility—especially when they are selenium deficient. In their research, 20 infertile men with AT were daily administrated with 83 µg of selenium as L-Selenomethionine via a commercially available supplement for six months. At the end of the treatment, an increased sperm concentration and sperm motility, a general better morphology, lower semen leukocyte concentration and percentage of spermatozoa with DNA fragmentation with respect to the pre-treatment data were observed [191].

Furthermore, considering the small number of studies available on this topic, random controlled trials on larger population should be carried out in order to determine whether selenium supplements could affect not only sperm parameters but also the success of fecundation.

The effect of selenium on female fertility has been reviewed by Rayman in 2012 [2]. However, very recent findings on mice models [192] showed increased ovary levels of SelK and SelM mRNA when the animals were administrated with inorganic or organic selenium. Furthermore, the production of blastocysts from oocytes was significantly higher in the Se-supplemented mice with respect to the Se-deficient group. Oxidative stress induced by excessive ROS or insufficient antioxidant protection in human oocytes and embryos can have detrimental effects on reproduction success [193].

In a cohort of 70 infertile women affected by occult premature ovarian insufficiency (OPOI), a 12-month long supplementation intervention with 200 μg/day of selenium and 400 IU of vitamin E was performed. OPOI patients have been characterized as having lower selenium plasma levels and increased ROS, something which might worsen the pathology in damaging primordial follicles and reducing anti-Mullerian hormone (AMH) levels. At the end of this intervention, an increase in anti-Mullerian hormone, antral follicle count and mean ovarian volume were registered without side effects [194].

The reported results appear to be consistent with the protective role hypothesized in the female reproductive system by antioxidant enzymes, including GPx1 and GPx3 [193,194]. By restoring the correct selenium levels via supplementation, a correct physiological activity of selenoproteins can be achieved, improving female fertility impairments.

Thus, also on the basis of these novel results, the effect of selenium on fertility represents a research field that surely deserves to be further investigated.

## 7. Bone and Skeleton Health

In humans, reduced serum selenium concentrations are associated both with increased bone turnover and reduced bone mineral density with a higher risk of bone disease. Thus, selenium is an essential nutrient for bone health and its role may be linked to the action of specific selenoproteins that maintain the redox cellular balance, protecting bone from oxidative stress and regulating the proliferation and differentiation of bone cells. An appropriate dietary selenium intake may have a potential role in preventing the development of osteoporosis.

Selenoprotein mutations and low selenium plasma levels are typical of skeletal diseases such as Kashin–Beck disorder and postmenopausal osteoporosis. Selenium levels were positively associated with the bone mass at femoral and trochanteric site and an adequate intake of selenium is inversely related to the risk of hip fragility fractures [195]. However, in this regard some authors have suggested that it would be more adequate to evaluate the bone mineral density (BMD) instead of the hip fracture related to selenium levels, because the fracture might be due to different causes than osteoporosis. Additionally, smoking status, drinking status, physical activity level, nutritional supplements, diabetes, hypertension, fibre intake, and calcium intake should be considered together in these patients, because they are all preventative or risk factors for oxidative stress that directly influences the bone health [196].

A recent cross-sectional study analysed the correlation between hair selenium level, which represents a reliable index to reflect long-term nutrition state, correlation and lumbar spine and femur BMD values in a population of adults. Individuals with lower hair selenium levels showed significantly lower BMDs with a greater increased risk of developing osteoporosis. The study suggests that measuring hair selenium levels may be an easy and quick strategy to be used with patients with osteopenia or osteoporosis in order to evaluate the most appropriate dietetic strategy [197].

In this context, previous studies have already highlighted the association between selenium serum concentration and lumbar spine BMD in Turkish women in post-menopause who had osteopenia or osteoporosis [198]. Furthermore, a positive association between general lower serum selenium levels and osteoporosis was pointed out. Individuals with lower selenium levels also showed lower femoral neck and lumbar spine BMD values [199]. A recent study compared plasma selenoproteins and selenium levels with BMD values in healthy aging European men. Intriguingly, selenoproteins and selenium levels were positively associated with BMD values, independently from the thyroid function [200].

A negative correlation between dietary selenium intake and the prevalence of osteoporosis was also found in the general middle-aged and older population in China. The BMD was detected at the phalanges with a compact digital RA system and the evidence extended both to men and women, showing a dose–response trend [196].

On the other hand, some authors have observed that an elevated selenium intake might negatively affect BMD in postmenopausal female subjects, depending on their calcium intake levels at time of measurement [201].

Very recently a randomised, double-blind, placebo-controlled trial [202] was conducted on 120 postmenopausal women with osteopenia or osteoporosis. Half of them were administrated with 200 μg/day of selenite. Urine N-terminal cross-linking telopeptide of type I collagen (NTx), a bone turnover marker associated with fracture risk, was measured and serum selenium and selenoproteins levels were also evaluated. With reference to the mechanism for selenium in maintaining skeletal health, the selenium ROS-reducing role and a possible contrast function against the pro-resorptive osteoclasts were considered. At the end of the intervention, while serum selenium and selenoprotein P increased from baseline, NTx did not change. However, the authors considered that the skeletal antoxidant function might actually be improved via supplementation but that NTx might be an inappropriate marker.

As already suggested [197], selenium plays a pivotal skeletal maintenance role and possesses antioxidant defence characteristics in the bone microenvironment, mainly in the form of selenoproteins, that are the essential selenium transporter and are expressed both in bone-resorbing osteoclasts and in bone-forming osteoblasts.

Low selenium and selenoproteins levels correspond to increased intracellular ROS concentrations that, via different mechanisms, inhibit osteoblastic differentiation of bone marrow stromal cells, contributing to the onset of osteoporosis. Additionally, selenium is critical in cell cycle progression and cell proliferation; a selenium deficiency results in G2 cell cycle arrest. Furthermore, it has been postulated that interleukin-6 (IL-6) and some other cytokines play a significant role in the pathogenesis of osteoporosis. Selenium can delay the onset and progression of the disease by exerting an inhibitory action on IL-6 and cytokine activities. Finally, since a selenium deficiency may increase the level of thyroid hormones in the blood, thus accelerating bone loss and osteoporosis genesis, this element is also indirectly related to skeletal health [197].

## 8. HIV

Selenium is implicated in the inhibition of viral expression, and in the delay of the progression of AIDS in HIV-positive patients [203,204]. HIV/AIDS is a major health priority worldwide and the development of efficient antiretroviral therapy increased the number of people living with HIV. Nutrient deficits, however, may interfere with the effectiveness of antiretroviral therapy by weakening the immune system that is directly dependent on selenium intake. This may be related not only to the role of selenium in immune functions, but also to its activity in modulating viral expression. Furthermore, as stated above, selenium is involved in the protection against oxidative damage, that is associated both with the chronic infection and with its treatment [205]. Several studies have highlighted that HIV infection is typically associated with increased ROS [205,206], with a consequent decrease of the major antioxidant nutrients, including selenium [205,207,208].

Antiretroviral drugs have been associated with increased oxidative stress and damage, especially in human aortic endothelial cells [209,210,211] which may cause the long-term development of atherosclerosis and coronary heart disease as a side-effect [205]. Thus, supplementation of antioxidants, including selenium, may be an important part of the therapy against the side effect of the treatment.

In HIV-infected children and adults, selenium deficiency has been associated with advanced immunodeficiency [205], disease progression and mortality [212,213,214]. The significant HIV-related mortality in situations of selenium deficiency demonstrate the importance of maintaining an adequate selenium status in HIV infected patients [213,215,216,217]. The beneficial effects of selenium on the immune system have been documented in animal and in human supplementation studies [205,218]. Selenium status influences HIV disease progression modulating cytokines expression, interleukin-2 production and the ability of phagocytic neutrophils and macrophages to destroy antigens.

As stated above, the correct functionality of antioxidant systems also depends on selenium, which also affects the production of tumour necrosis factor-α (TNF-α). Plasma selenium levels were inversely associated with TNF type II receptors in HIV-positive patients [205]. Selenium supplementation in HIV-positive patients has shown benefits on biomarkers of disease progression, morbidity and mortality [205], reducing the viral replication and increasing Glutathione peroxidase activity in latently HIV infected T-lymphocytes [219,220]. Furthermore, the glutathione peroxidase and thioredoxin reductase 1 activity in macrophages, normally decreased after HIV infection, improved with selenium supplementation [221]. This was speculated to be linked to the activity of glutathione and thioredoxin systems. Indeed, selenium supplementation improved the expression of GPx1 and TrxR1 in HIV patients (often deficient for selenium and consequently for selenoenzymes) and also inhibited HIV transcription and replication. This was probably due to the lower oxidative stress levels and decreased expression of the Nuclear factor kappa-light-chain-enhancer of activated B cells (NF-κB) and pro-inflammatory cytokines, which have a pivotal role in the HIV infection [205,221].

Several studies about selenium status and HIV progression observed a direct association between low plasma/serum selenium concentration or erythrocytes GPx1 activity, and a reduced count of CD4+ lymphocytes, with a greater HIV progression and mortality. An adequate selenium status in HIV patients may increase immune defences, thus improving general health and reducing the hospitalization for opportunistic infections [1]. In this context, some randomised controlled trials highlighted the benefits deriving from selenium supplementation in HIV patients, with a significant decrease in hospital admissions [2].

In a 24-month double-blinded, placebo-controlled, randomized clinical trial on 300 Highly Active Anti-Retroviral Therapy (HAART) patients, the effect of selenium on the rate of CD4 glycoprotein decline, viral suppression, and morbidity were evaluated. The rate of CD4 decline was reduced by 43.8% in the subjects that were administrated with 200 µg of selenium a day, with overall benefits for the immune system [222]. In this regard, another randomized, double-blind clinical trial involved 878 HIV-infected, HAART adult subjects. A daily supplement of 200 μg selenium + vitamins significantly reduced the CD4 count decrease with respect to the placebo group, with a minor risk for the clinical manifestations of AIDS-related complications and death.

These results confirm the important role of selenium, even if administered along with a multivitamin, in the maintenance of the immune system [223]. Specific trials on HIV-infected pregnant women show neither amelioration on the CD4+ cell count [217] nor decrease in the preterm delivery [224]. However, this may be due to the poor baseline nutritional status of the considered patients.

## 9. COVID-19

The novel coronavirus Severe Acute Respiratory Syndrome-coronavirus-2 (SARS-CoV-2), causing the coronavirus disease COVID-19, is a dangerous coronavirus responsible for a global pandemic and severe public health crisis. Indeed, the severity of the COVID-19-related worldwide pandemic situation has surpassed the past acute respiratory syndrome coronavirus of 2003 (SARS-CoV-1 or SARS-CoV) and Middle East respiratory syndrome coronavirus of 2012 (MERS-CoV), which were limited to more restricted areas [225,226]. At time of writing, SARS-CoV-2 has caused over 293 million infections and it has been responsible for over 5.4 million deaths [227].

Despite the recent production of specific vaccines, the global threat deriving from COVID-19 to human health and economy persists, especially considering the diffusion of new variants to the original viral strain. Fast, reliable and safe measures for reducing infection risk, suppressing virulence development, strengthening the immune system, and supporting recovery are needed. Selenium may play a relevant role for most of these issues, having a wide range of protective functions, primarily a complex immune-modulator action mediated by specific selenoproteins [228,229].

Many studies have documented that selenium deficiency can cause an increased host-susceptibility to RNA viral infections and more critical disease outcome up to mortality [229,230].

An analysis of very recent literature about the relationship between SARS-CoV-2 and trace elements provided direct evidence for an association between selenium and COVID-19. An adequate selenium intake is essential for resistance to viral infections, boosting the immune function and reducing inflammation that favours the onset of the infection. Observational studies have shown that nutritive supplements administered at an early stage of the infection were important for enhancing the host resistance against RNA viral infections, such as COVID-19. In fact, selenium deficiency supports mutations, replication and the virulence of RNA viruses. Selenium has a wide spectrum of pleiotropic effects in COVID-19 disease, restoring the host antioxidant capacity, reducing apoptosis and the effect of SARS-CoV-2 on endothelial cell damage as well as on platelet aggregation. Low selenium status is a common indicator in patients at risk to develop severe COVID-19-related syndrome, especially in vulnerable, obese and elderly patients who are particularly susceptible to high levels of inflammatory cytokines. Selenium might thus represent a game changer in the global response to COVID-19 [228,229].

**Figure 6:**
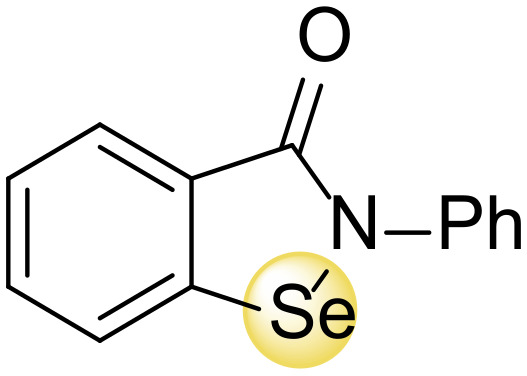
Structure of ebselen.

When the SARS-CoV-2 virus enters the lung cells, it exploits the cell structures, interfering with metabolic and physiologic processes. Oxidative stress response arises from such activities and the budding of the virion from host cells further disrupts the cell membrane, causing cell lysis, an enhanced ROS production and the activation of inflammatory signalling pathways. The level of oxidative stress in COVID-19 can be linked with the severity of the disease itself (extension of tissue damage and hyperinflammation). The redox activity of selenium species, particularly low-molecular selenium compounds such as methyl-selenol, dimethyl-selenides, (mostly achieved in human body by high selenium intake), selenium nanoparticles, and other selenium-containing molecules, can stop the viral life cycle by interrupting its replication and transcription. In particular, these processes are due to 3C-like protease (3CL^pro^) or M^pro^ (formally known as C30 Endopeptidase), the main SARS-CoV-2 protease that allows viral maturation within the host. Ebselen (Figure 6) was shown to directly inhibit M^pro^ activity, by covalently binding the sulfhydryl group of the Cys145 residue in the catalytic dyad [228,230].

Papain-like protease (PL^pro^) is another enzyme that SARS-CoV-2 uses to antagonize the host’s antiviral innate immune response. Ebselen was found to highly inhibit PLpro through a covalent binding with the sulfhydryl group of the Cys112 residue in the catalytic triad [228].

Some authors have suggested that the selenium protective action mechanism could also involve an increased resistance toward virus-induced cytokine release syndrome. Both selenoproteins and redox-active selenium species (such as ebselen and related GPx mimics) could be involved in slowing down virus-triggered oxidative stress, abnormal inflammatory responses and immune-system failure, thus improving the prognosis of SARS-CoV-2 infection [228].

The administration of antioxidant seleno-derivatives may indeed be pivotal in contrasting the onset or ameliorating the clinical course of COVID-19 infection [228,230]. A nutrition intervention with an adequate supplementation may be protective or coadjutant in COVID-19, especially in vulnerable groups of populations or high-risk areas, such as developing countries [225].

An association of mortality risk with selenium deficiency in COVID-19 patients has been highlighted. Moghaddam et al. conducted a cross sectional study on 39 COVID-19 patients in a German hospital. The researchers found that selenium plasma levels were significantly higher in surviving, with respect to non-surviving, COVID-19 patients [231]. Furthermore, a significant, positive, linear association was found between the cure rate of Chinese patients with COVID-19 and regional selenium status [228].

As aforementioned, selenium seems to play a role in COVID-19 disease aggressiveness and positive convalescence. Thus, supplementation could be considered in the most severe cases and in selenium-deficient patients. Although the causality mechanism is still unclear, preliminary observational studies also revealed that selenium status analysis in COVID-19 patients could provide useful diagnostic information, even if the causality mechanism is unknown. Intervention studies should be set in order to clarify the relationship between selenium and SARS-CoV-2 disease, and to define possible preventive measures or adjuvant treatments via selenium supplementation [231].

Heller et al. proposed a composite biomarker including Selenoprotein P and zinc as a reliable indicator of survival in COVID-19 and suggested that a personalized supplementation of selenium and/or zinc may support convalescence [232]. COVID-19-associated inflammation has been linked to a reduced expression of many selenoproteins, including glutathione peroxidase, thioredoxin reductase and those involved in controlling endoplasmic reticulum (ER) stress and the expression of interleukin-6 (IL-6) in SARS-CoV-2 infected cells. This is further accentuated in obese patients, who also generally show lower selenium status, for whom dietary selenium supplementation may help to alleviate the respiratory and inflammatory clinical symptoms [233].

Selenium also plays a role as an NF-κB inhibitor, with consequent immune-modulation and anti-inflammatory action [229]. The cytokine release has a negative effect on COVID-19 and, especially in elderly people, selenium deficiency is correlated with higher circulating inflammatory cytokines. On the other hand, selenium adequacy prevents excessive cytokine activation in infections and inflammatory models. In some cases, high doses of selenium contributed to an increase in the adaptive immunity and moderation of the release of inflammatory cytokines by the innate immune system [228].

A connection between more-than-adequate selenium intake/status and higher cure rate has been highlighted. Daily doses of 1 mg selenium (in the form of selenite) have already been used in sepsis and critical care applications. Taking into account the above, preliminary results have suggested that the use of selenium should be clinically tested, preferably in randomized controlled trials [228].

**Figure 7:**
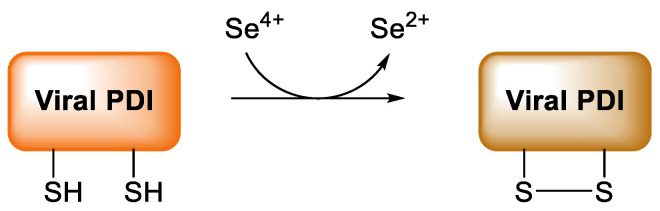
Se(IV)-promoted oxidation of thiol functions of viral protein disulphide isomerase (PDI).

Selenite tetravalent cation (Se^4+^) can be reduced to divalent cation (Se^2+^), acting as an oxidant. This oxidizing capacity has important implications for its antiviral property. Selenite quickly reacts with sulfhydryl groups in the active site of viral protein disulphide isomerase (PDI), oxidizing and inactivating this enzyme according to the reaction showed in Figure 7. In this way the viral hydrophobic spike can no longer perform the exchange reaction with disulphide groups of cell membrane proteins, and consequently the virus cannot enter the healthy cell cytoplasm [234,235].

On the basis of these results, it seems reasonable to speculate that sodium selenite, a rather inexpensive and readily available molecule, could represent a potential agent for the prevention of viral infections including Coronavirus, according to the mechanism already suggested for other infections such as Ebola, Polio and Influenza [235]. Considering that the acute infection phase in COVID-19 is only a few weeks long in typical cases, it may be reasonable to consider the same supra-nutritional selenium administration for such a short time, in order to deliver benefits to patients with moderate-to-severe symptoms without toxicity risks. The whole potential of this strategy is a preliminary suggestion that would need to be tested clinically to be validated, preferably in a randomized, controlled trial in large cohorts [228,230].

## 10. Cognitive Decline and Alzheimer’s Disease

Serum selenium concentrations decline with age and low selenium concentrations might be associated with age-related declines in brain function, plausibly due to decreases in antioxidant activity [236,237]. In this context, studies in areas with low selenium content in soil, such as some regions of rural China, have demonstrated that lower dietary selenium levels are associated with poorer cognitive function [238].

Kesse-Guyot et al. analysed the data of 4447 participants aged 45 to 60 years from the French SU.VI.MAX. study. They reported that the administration of an antioxidant supplement including ascorbic acid, vitamin E, β-carotene, selenium, and zinc for approximately eight years was associated with higher episodic memory and semantic fluency test scores. However, the independent contribution of selenium to the general observed effects cannot be determined [239]. Similarly, the InCHIANTI cohort study involved 1012 participants aged 65 years or older, whose coordination performance were assessed. The lower selenium levels were significantly associated with decreased performance in neurological tests [240]. In the French EVA cohort of 1166 people aged 60–70 years a significant increase in the risk of cognitive decline was recorded over four years in participants with low baseline plasma selenium [237]. Restoring correct levels of selenium in the body through the diet—via administration of one Brazil nut per day, containing about 288 micrograms of selenium, for six months—improved the cognitive performance of patients [241].

A cross sectional study including 2016 participants with adequate selenium status provided the first evidence of a sex difference in the association between selenium status and cognitive performance in older adults. Particularly, a positive association between blood selenium concentration and cognitive performance was found in males but not in females [242].

Alzheimer’s disease (AD) was described for the first time in 1906 and, despite years of study, the aetiology of this disorder remains poorly understood. The disease is characterized by the production of extracellular amyloid plaques that spontaneously aggregate into oligomeric forms, and by the presence of intracellular neurofibrillary tangles, formed from aggregates of the protein tau within the large pyramidal neurons [243]. The current treatment of Alzheimer’s disease is only mildly effective in maintaining cognitive function. Early research has suggested that different forms of selenium may be effective in the prevention or the treatment of this disorder. Selenium, alone or combined with vitamin E, has been proposed for treating or preventing Alzheimer’s disease, primarily because of its antioxidant properties [243]. Although the selenium concentration in the brain is not as high as in other organs, selenium is preferentially retained in this organ under conditions of low selenium intake and it is essential for proper brain function [244]. Owing to the high oxygen utilization and the abundance of oxidizable metals, the brain is particularly reliant on antioxidant mechanisms that include several selenoproteins and seleno-compounds [243,245]. In this regards, Seleno-L–methionine was demonstrated to be protective against oxidative stress and against toxicity from β-amyloid in cell culture and in rodent models [243]. Sodium selenite can inhibit amyloid production by decreasing γ-secretase activity, while sodium selenate can reduce neurofibrillary tangle formation [243,246,247].

In Alzheimer’s disease, oxidative damage of proteins, lipids, and nucleic acid is particularly relevant in areas of amyloid plaques and in cells with neurofibrillary tangles [248]. Several selenoproteins are also important for the mitigation of oxidative stress in Alzheimer’s patient’s brains; particularly GPx1, GPx4 and TrxR1 work synergically for the reduction of peroxides, free radicals, and oxidized biomolecules. Moreover, Gpx1 may act as a neuromodulator, impacting on neurodegenerative and neuropsychiatric disorders (not only AD but also PD, schizophrenia and bipolar disorders) as very recently discussed by Sharma et al. [249]. Other selenoproteins such as SelP, SelW and the ER-resident selenoproteins K, T, M (SelK, SelT, SelM) have been suggested to play pivotal roles in the brain [250]. Selenoproteins in the brain may act as antioxidants using either glutathione or thioredoxin as substrates. Ref. [243] Recent studies focused on the role of SelP in Se-delivery to neurons, antioxidant activity, cytoskeleton assembly, chelation of redox-active metals (copper and iron), and interaction with misfolded proteins (amyloid beta and tau protein). Furthermore, a possible involvement in glial activation and brain cholesterol metabolism, related to signalling, has been hypothesised. Future animal model and human-based studies are needed to clarify these topics [251]. The exact mechanism that implicates SelP in Alzheimer’s disease has to be further investigated and discussed. SelP knock-out has already been shown to increase neurotoxicity caused by amyloid peptides [252]. Additionally, there is evidence for the role of selenoprotein P as a signalling molecule associated with the neuronal mechanism of long-lasting memories [243]. Thus, a potential role of SelP in the formation of amyloid plaques and neurofibrillary tangles, as well as in memory pathways, has been hypothesized. In this regard, SelP may behave as a protective agent against AD-related oxidative stress [243,253]. Post-mortem studies on tissue from Alzheimer’s patients has highlighted that SelP, normally increased in the ageing brain, showed a particularly significant increase in amyloid-beta plaques, neurofibrillary reticles and cerebrospinal fluid [253,254]. According to several studies, SelP elicits a transport function to the brain—that is targeted via specific receptors such as ApoER2 [255,256,257]—preserving its function under conditions of selenium deficiency. Early studies have shown that SelP prevents Aβ and hyperphosphorylated tau aggregation and toxicity and that it also interacts with redox-active metals in the brain, such as Cu, Fe, and Hg. SelP showed antioxidant enzymatic activity, signalling functions, and provides selenium to neurons and glial cells for the synthesis of antioxidant selenoproteins that mediate the redox–stress response [251].

At the present level of knowledge, there is no rationale for supplementing selenium to population groups where selenium status and selenoproteins concentration are already adequate. No effects or even symptoms of toxicity were observed when supplementing selenium in a population that already had an adequate selenium status [251].

Future studies should be set carefully considering the selenium levels of participants at baseline, in order to highlight the real potential effects of selenium supplementation in preventing cognitive decline and, possibly, AD in the general population. Additionally, selenoproteins could be involved in the regulation of ER-Ca^2+^ flux and balance at the synaptic level and in the degradation of the uncorrected folded protein [250].

With reference to the metal chelation role of selenium, which indirectly protects the brain from oxidative stress, recent investigation in in vitro models focused on the Aβ aggregation process. Selenium nanoparticles stabilized with chitosan (Ch-SeNPs) inhibited the metal-induced Aβ aggregation, also showing a significant disaggregation capacity of Aβ fibrils, and reducing their length and width [258].

Proper folding of proteins in the endoplasmic reticulum (ER) is essential for their intended function, and errors in this process require a correction via the endoplasmic-reticulum-associated protein degradation (ERAD) system. Early studies have already suggested an important role for ER stress in Alzheimer’s disease, indicated by the presence of specific markers in Alzheimer’s patients’ brains [243,259]. ER stress can be triggered by the presence of extracellular amyloid β and, in turn, can promote the formation of neurofibrillary tangles [260]. In this context selenoprotein S (SelS, VIMP, or SEPS1) has an important role in ERAD and, therefore, a possible preventative role in neurofibrillary tangles formation [261]. On the other hand, considering that calcium has important roles in neuronal signalling, survival, and cell death, loss of calcium regulation may be an important part of the pathology of Alzheimer’s [262,263]. A growing number of selenoproteins have been implicated in regulating calcium flux from ER, such as selenoprotein M that, as previously reported, alters ER calcium signalling in neurons and protects neurons from oxidative stress [264]. Furthermore, selenoprotein N (SelN or SEPN1) expression alters calcium signalling through the calcium-sensitive ryanodine ER receptors [243]. Selenoprotein T can also alter calcium release from ER deposits in neuroendocrine cells in response to the Neuropeptide polyadenylate cyclase-activating polypeptide (PACAP). Thus, the selenoprotein family appears to have significant importance in ER calcium regulation and homeostasis [265].

Despite the potential importance of selenium in AD investigations about selenium levels in AD patients are very limited due to the difficulties and variability in living environments and dietary states. This is probably also the main reason for the conflicting and inconsistent results currently available [266]. A recent systematic review and meta-analysis of 14 studies [267] found a significantly lowered selenium status in AD patients’ brains, with the lowest values in the temporal and hippocampal regions, which are pivotally involved in the memory processes. The decreased selenium levels in these areas may play an important role in the pathophysiology of AD, by also impairing the proper expression and activity of the aforementioned selenoproteins.

Mouse model studies have provided initial evidence about the beneficial role of selenium supplements in AD. Such supplements play a significant role in maintaining correct selenium levels and selenoenzymes activity, which are believed to slow down the progression of symptoms. Ebselen ameliorated memory impairment, hippocampal oxidative stress, apoptosis, and cell proliferation in a mouse model of induced Alzheimer’s disease [268]. Selenomethionine restored the structural and functional plasticity of synapses in AD mice [269]. Selenium-based supplements proved to be efficient against neurodegeneration. For example, ebselen was shown to modulate oxidative stress and to reduce Aβ and p-tau, improving postsynaptic density in AD models. Furthermore, transgenic AD mice treated with ebselen showed improved results in spatial and memory tests [268,270].

Normally AD patients show significantly lower selenium levels in plasma than healthy people; this may be related to the disease onset through the mechanism discussed above. A multiple linear regression analysis showed that frequent consumption of a nutritional pattern including bread, butter, coffee, cheese, and tinned fish may be associated with increased selenium concentration in the serum of patients with Alzheimer’s disease. An adequate consumption of dietary antioxidants including selenium may be a preventive factor [271].

A very recent study on 40 AD patients in different clinical stages and 40 healthy controls found high selenium levels in nail and hair samples from AD patients. The authors hypothesised that the higher selenomethionine in nails and hairs corresponded to a lower selenocysteine concentration in the brain, thus explaining neurodegeneration as a consequence of the impairment of active selenium forms [266]. According to Vinceti et al., past case-control studies do not allow a reliable assessment of the role of selenium exposure in AD aetiology since they considered data about peripheral selenium exposure (e.g., toenail, hair, serum or plasma levels) and not central nervous system indicators such as cerebrospinal fluid [272]. In this regard Vinceti et al. recently performed a study focused on the analysis of cerebrospinal fluid (CSF). The results of this study showed that AD risk is inversely correlated with inorganic selenium species and with the organic form bound to selenoprotein P in the CSF. On the other hand, some previous studies had shown no significant differences in CSF and serum selenium levels between AD-patient and control groups [273,274,275]. Cardoso et al. examined levels of selenium and selenoproteins in serum and cerebrospinal fluid (CSF) in a pilot study involving 40 AD cases [276]. The patients were randomized to placebo, nutritional (0.32 mg of sodium selenate, three times/day), or supra-nutritional (10 mg, 3 times/day) groups. After 24 weeks of treatment serum and CSF selenium levels were measured and compared against cognitive outcomes. Sodium selenate supplementation at a high or supra-nutritional dose induced an increase in selenium uptake into the CNS, with elevation in CSF selenium and corresponding to subtle but significant improvement in Mini-Mental Status Examination test (MMSE) scores. Although individual variation in selenium metabolism must be considered, along with the increased mortality in healthy elderly subjects deriving from long-term supplementation, the authors concluded that selenium should be considered for potential benefits in AD [276]. In this context, a probiotic (*L. acidophilus*, *B. bifidum*, and *B. longum*) and selenium (200 mg/day) co-supplementation for 12 weeks to patients aged 55 to 100 with AD was also found to improve cognitive function and some metabolic profiles with respect to the placebo group [277].

A synthetic overview of recent studies focusing on the role of selenium in Alzheimer’s diseases is reported in Table S2 (see Supplementary Materials).

## 11. Parkinson’s Disease

Parkinson’s disease (PD) is a neurodegenerative disorder characterized by the loss of pigmented dopaminergic neurons in the substantia nigra, and by the simultaneous presence of intraneuronal protein inclusions called “Lewy bodies”. Dopaminergic neurons are particularly vulnerable to oxidative stress, mainly due to their accumulation of iron ions with advancing age. Oxidative stress has been described as a major contributor to the development and progression of neurodegeneration at the cellular level [278,279,280,281,282].

Oxidative stress, in fact, damages intracellular organelles, in particular the mitochondria, impairing neuronal energy metabolism and, as a consequence, neurotransmission and neuritogenesis. Mitochondrial dysfunction trigger apoptosis, calcium release, and opening of mitochondrial permeability transition pores (mtPTP), leading to the death of neurons, including specific dopaminergic neurons. In turn, the imbalance of dopamine metabolism contributes to ROS production. Dietary antioxidants, by interacting with ROS, have a significant role in the termination of oxidative chain reactions [282]. In this regard, the maintenance of an adequate antioxidant nutritional status may be a strategy in the prevention or slowing down of PD.

The biological function of selenium is implemented through selenoproteins, which contain a selenocysteine residue in the active site. Selenium is particularly uptaken by the brain tissue, where it plays different functions, one of the most important of which is the antioxidant function. Selenium deficiency can be a risk factor for diseases associated with oxidative stress, including PD [283]. However, despite these premises, it is difficult to state a cause-and-effect relationship between selenium and the pathophysiology of Parkinson’s disease. Indeed, the general impairment of the motor system is associated with the overall malnutrition condition. Therefore, such impairment is not only related to selenium but also to other microelement deficiencies that could affect the progression of the disease [240,284]. A number of recent studies in animal models have suggested that a selenium deficiency could contribute to a greater vulnerability to oxidative stress by dopaminergic neurons. In particular it was observed that a preparatory treatment based on selenium as hyposelenite, before the exposure to parkinsonian neurotoxins, could decrease dopamine depletion of the striated area in a dose-dependent way [285].

On the other hand, a very recent systematic review and meta-analysis of 56 case-control studies highlighted that selenium levels in serum or plasma of PD patients were similar to the controls data; additionally, cerebrospinal fluid (CSF) levels were considerably higher in PD patients [286]. These findings were also confirmed by a later meta-analysis of 11 studies by Zhang et al. [287].

According to Adani et al., a selenium overexposure in the central nervous system might even be a cause of PD onset rather than a protective factor. Notwithstanding the multiple beneficial properties of selenium for the human body (antioxidant activity, regulation of Ca^2+^ channels, modulation of neurogenesis) it may also exert some adverse effects, especially in case of overexposure, that may lead to neurodegeneration, directly via alteration of the mRNA expression of dopamine receptors [286].

As aforementioned for the case of AD (vide supra), the use of CSF samples data instead of blood and plasma data about selenium levels in PD patients was actually supported in recent years by different authors, in order to perform more reliable studies [286,287,288]. An attempt to determine the different selenium species present in CSF of PD patients was carried out by Maass et al. [289]. Seventy-five PD patients and 68 age-matched controls were enrolled; eight different selenium species were detected in the CSF samples. Only selenoprotein P, human serum albumin-bound Se (Se-HSA), selenomethionine (Se-Met) and an unidentified Se-compound (U2) were shown to have significant quantification values. No significant differences between the cases and controls were found. According to this study, the role of selenium neurotoxicity in the onset of PD pathology may thus be not so relevant as previously hypothesized (Table S3, Supplementary Materials).

An additional interesting point is related to the gut-brain axis; 21.5% of gut microbiota-sequenced bacteria express selenoproteins [288]. It has been demonstrated that alterations in the human microbiome represent a risk factor for PD [290]. Thus, the gut-brain axis may have a strong implication in the pathogenesis of Parkinson’s disease that is worthy of further investigation.

## 12. Schizophrenia, Anxiety and Depression

Schizophrenia is a severe neuropsychiatric disorder occurring in childhood, adolescence or adulthood. It is characterized by a heterogeneous mixture of positive, negative and cognitive symptoms such as flat affect, catatonia, impaired attention and memory, hallucinations. This condition is influenced by multiple environmental and genetic risk factors. Several studies have highlighted that nervous system damage possibly connected to schizophrenia pathophysiology may be associated to oxidative stress [291]. In fact, ROS can damage neurons by lipid peroxidation, protein carboxylation, DNA strand breaks, and alter cell signalling cascades which regulate several neurotransmitter systems, resulting in altered dopaminergic, glutamatergic, and GABAergic neurotransmission. Considering that glutathione peroxidases, thioredoxin reductases, and iodothyronine deiodinases are critically involved in the protection mechanisms operating against oxidative stress, an impaired biosynthesis and function of these selenoproteins may contribute to the pathogenesis of schizophrenia [292].

Decreased levels of Glutathione (GSH) in schizophrenic patients were first noted in 1934 [293] and several following studies have documented a correlation between low GSH levels and schizophrenia [294,295] also providing genetic evidence for a link between schizophrenia and impaired GSH synthesis [296]. Significantly reduced GPx activity has been reported in groups of patients with schizophrenia receiving treatment with antipsychotic medication [292,297]. Furthermore, an inverse relationship between blood GPx activity and structural assessments of brain atrophy has been observed in a population of patients with chronic schizophrenia, suggesting a potential link between redox dysregulation and neurodegeneration. Circumstantial evidence has also suggested that altered function of the mitochondrial selenoprotein thioredoxin reductase 2 (TrxR2) may contribute to schizophrenia [292].

Intriguingly, among the United States population, higher incidences of schizophrenia have been reported in States with low levels of selenium in the food chain [292]. In addition, impaired selenium transport was previously hypothesized to be a risk factor for a subtype of schizophrenia characterized by negative symptoms. This is supported by the reduced platelet and erythrocyte GPx activity in schizophrenic patients [292]. Several studies have also highlighted that dopaminergic signalling was related to dietary selenium intake, suggesting a potential indirect relationship with schizophrenia. Indeed, dietary selenium deficiency elevates and extends high potassium-induced dopamine release in the striatum and increases the turnover rate of dopamine in the substantia nigra, prefrontal cortex, and hippocampus [292]. Furthermore, selenium deficiency in rat model up-regulates both tyrosine hydroxylase and dopamine transporter mRNAs in nigrostriatal neurons, with concomitant increases in dopamine synthesis and uptake [298].

Conversely, dietary selenium supplementation reduces the activity of the dopamine catabolic enzyme monoamine oxidase (MAO) that also generates peroxides in a process coupled with the enzymatic activity of GPx1 [292,299]. Collectively, these findings suggest that dietary selenium modulates the turnover and metabolism of dopamine, which may profoundly affect the pathogenesis of schizophrenia.

Recently, a study conducted on 21 schizophrenic patients showing low baseline serum selenium levels highlighted the effect of selenium supplementation. Patients were administrated with 60 μg of selenium a day, in the form of selenium-enriched yeast, via a commercially available supplement. After three months of treatment the serum selenium levels increased and the patients showed enhanced appetite and improved memory [300].

In 2020 Ma et al. reported a case-control study (99 cases, 99 controls) investigating the potential association existing between some essential elements, including selenium, and the risk of schizophrenia in China [291]. Consistent with the results obtained by Li et al. [300], a decreased selenium concentration in serum was significantly associated with the risk of schizophrenia and disease severity. According to the authors, the physiological suppression of oxidative stress by selenoproteins may exert a neuroprotective role [291]. Additionally, the role of selenium in the dopamine pathways was pinpointed. In the same study, the authors also highlighted that serum selenium concentration was positively correlated with the serum levels of several metabolic biomarkers of glucose metabolism (fasting blood glucose), lipid metabolism, (triglycerides, total cholesterol), liver function (aspartate transaminase, alanine transaminase, albumin and total protein), renal function (blood urea nitrogen, creatinine, uric acid), and blood cell count (red blood cells white blood cells, platelets and haemoglobin). These markers were significantly altered in schizophrenic patients with respect to healthy controls, suggesting a possible correlation with large-scale metabolic disorders in schizophrenic patients [291].

On the other hand, a 2020 review and meta-analysis of 10 studies with a total of 1784 participants compared blood selenium levels in patients with schizophrenia and healthy controls. The results showed no significant association between schizophrenia and blood selenium levels [301].

In the last years some studies have also showed that selenium intake and plasma levels could be inversely associated with depression [302,303,304,305] and anxiety [306]. This association was also demonstrated for patients with euthyroid nodular goiter, independently from the thyroid function [306]. In this context, an “omnivore” nutritional pattern including high selenium levels administrated in a cross-sectional study resulted in significant protection against depression, psychological distress and anxiety [307].

## 13. Type-2 Diabetes

Type-2 diabetes (T2D) is characterized by defective insulin secretion and/or insulin resistance. Currently, the relationship between selenium and type-2 diabetes remains only partially understood and mainly controversial. Some early case-control and prospective studies have associated higher selenium levels in the body with a reduced diabetes prevalence and lower hyperglycemia risk. However, other studies have suggested a non-relevant effect of selenium on insulin metabolism. For example, 400 subjects were involved in a randomized, placebo-controlled trial receiving 200 μg/day of selenium or a placebo. After 2.9 years of intervention, selenium showed no effect on insulin sensitivity or β-cell function (esteemed from HOMA2-%β or HOMA2-%S) compared with the placebo group [308]. On the other hand, in different cohorts, high serum selenium concentrations have been correlated with an increased prevalence of diabetes, higher fasting plasma glucose or no effects at all. Results of the first studies have been reviewed by Rayman in 2012 and, therefore, are not covered by this review [2]. Later systematic reviews and meta-analyses [309,310,311] agreed in finding a consistent positive association between the exposure to selenium and the increased risk/prevalence of T2D. A synthetic overview of recent studies focusing on the role of selenium in T2D is reported in Table S4 (see Supplementary Materials).

Dubey et al. included selenium in their complex analysis about the relationship between trace minerals and diabetes. The authors highlighted inconsistent results between different studies and claimed the necessity of further investigation [312]. The most recent epidemiological and interventional trials focused their attention on the association between high selenium intake and increased risk of T2D [313].

In a cohort of 655 subjects serum selenium status was suggested to be correlated with obesity and type-2 diabetes, plausibly due to its effects on signalling pathways [314]. Liao et al. analysed data of 2903 participants from the US National Health and Nutrition Examination Survey (NHANES), highlighting that T2D patients have higher selenium levels compared with healthy subjects. Notably, the risk association was particularly higher in younger women [315]. Similarly, in a large population of Italian adults from a Moli-sani study cohort (21,335 subjects), a high dietary selenium intake was recently associated with increased risk of hospitalization for diabetes [316]. In our opinion, some of the apparently contradictory findings discussed above might be explained considering a U-shape association between selenoprotein levels and type-2 diabetes risk, depending on the baseline level of selenium intake. The hypothesis of a U-shaped association between selenium status and supplementation with glucose metabolism is reinforced by results of a recent study on 491 volunteers aged 60 to 74 years from the Denmark PRECISE cohort [317]. They were randomly assigned to treatment with 100, 200 or 300 μg selenium/day in the form of selenium-enriched yeast or placebo-yeast. HbA1c was measured at baseline, at six months, and after two years of selenium supplementation. At the end of the study, HbA1c had decreased significantly in all treatment groups. Compared with placebo, small beneficial changes in HbA1c were observed after six months in the lowest dose level of Se supplementation groups (100 μg/day). Further research is needed to clarify the optimal range of selenium intake and status for minimizing the potential adverse effects on glucose metabolism while preventing type-2 diabetes [317].

The biochemical mechanisms that underlie the correlation between selenium and insulin resistance/diabetes are not clearly understood. Selenoproteins may exert their role acting on the insulin signalling as well as on oxidative stress modulation [309,310]. A number of studies, also involving animal models in which the expression of selected selenoproteins has been genetically altered, have been conducted in order to investigate the role of selenoproteins on glucose and lipid metabolism-related diseases.

Both deficiency and high levels of selenoproteins can promote diabetes development. Different selenoproteins are involved in the regulation of glucose homeostasis, although the mechanism is still not fully understood. SELENOT is involved in blood glucose homeostasis as well as in maintaining the structural integrity of the endocrine pancreas. SelT KO in pancreatic β-cells of mice led to impaired glucose tolerance, due to altered insulin synthesis or release; the islet morphology was altered and smaller-sized in knockout compared with wild-type mice [8]. Both knockout and over-expression of GPx1 in transgenic mice may induce diabetes. An appropriate expression and activity of this selenoprotein is pivotal for controlling redox balance and glucose and lipid metabolism. GPx1 deficiency results in an excessive ROS accumulation, that inhibits gene expression or protein production of key transcriptional factors, leading to lowered islet β-cell mass and reduced insulin synthesis, and insulin secretion with a T1D phenotype. On the other hand, in certain tissues a physiological level of ROS is essential to control protein phosphatase involved in the insulin signalling pathway. In those tissues an uncontrolled reduction of intracellular ROS by a higher-than-physiological GPx1 expression might desensitize insulin signalling; this desensitization leads to insulin resistance in the context of T2D [318].

The binding of insulin to its receptor initiates a cascade which induces a mild controlled oxidative burst, in which H_2_O_2_ is involved. GPx1, by removing hydrogen peroxide, might thus interfere with this pathway [319]. This was preliminarily confirmed by experiments on transgenic mice: those over-expressing GPx1 showed insulin resistance and hyperinsulinemia, while knockout models exhibited improved insulin sensitivity [318,320,321]. Confirmation in humans was derived from the insulin resistance already observed in pregnant women in association with increased erythrocyte GPx1 activity [320] and also from enhanced insulin sensitivity in patients with global genetic selenoproteins deficiency [322].

On the other hand, an excessive oxidative stress may impair the correct function of pancreatic β-cells and, in this case, antioxidant selenoproteins such as GPxs may have a protective role. The overall insulin regulation thus requires fine tuning. Other selenoproteins are also involved in glucose metabolism [317].

SelP instead inhibits insulin signalling by inactivating the Adenosine monophosphate-activated protein kinase (AMPK), a positive regulator of insulin synthesis in pancreatic β-cells [1]. Clinical studies have shown that higher SelP concentrations are associated with insulin resistance and type-2 diabetes, glycated A1C haemoglobin and fasting plasma glucose [323].

In this context, C57BL/6J mice treated with intraperitoneal injections of purified human Selenoprotein P showed glucose intolerance and insulin resistance. The results of the study suggest that SELENOP impairs insulin signalling in the liver and skeletal muscle and induces glucose intolerance in vivo [324]. Considering that, as mentioned above [323], serum SelP levels are also elevated in people with type-2 diabetes compared with normal subjects, the study suggests that the secretory protein SelP could represent a target to develop new therapeutic strategies for the treatment of insulin resistance-associated diseases.

## 14. A Gender Medicine Approach for Selenium-Related Health?

None of the 25 human genes encoding selenoproteins is located on the Y-or X-chromosome, however women and men differ in several aspects of selenium metabolism. Both selenoproteins and Se-binding protein activity are regulated in a sex-specific way. SelP and ApoER2 are abundantly expressed in male testes while they are marginally present or absent in female ovary and uterus. This pronounced difference may be significant for the differential selenium retention and use in males and females. Currently, the general available data highlight that males are more responsive to acute changes in Se-supply, responding with faster kinetics and stronger amplitude. Men also seem to be more sensitive to the toxic effect deriving from an excessive selenium intake [325]. For these reasons, sexual dimorphism should always be considered when analysing the results of both observational and intervention studies, together with baseline selenium status and the reference population.

For example, with reference to the cancer risk, the first studies have already showed that the preventive effects were different in men and women, being more pronounced in males especially for lung, colorectal and stomach cancer [326,327,328,329]. More recently, a different correlation was found for bladder cancer, whose risk seems to be inversely associated with selenium concentration in the body in women, but not in men [325,330,331]. With reference to colorectal cancer, different studies have shown opposite conclusions on the best protection to men or women from selenium supplementation [332,333]. Additionally, subfertility and mortality in sepsis have been claimed to be mainly observed in males rather than females. Actually, the male reproductive system is more strictly dependent on selenium than the female one; women were underrepresented in the trials for supplementation in sepsis patients.

Selenium-dependent health effects in thyroiditis—and especially Hashimoto’s thyroiditis (HT)—are described only in females as are the associations between selenium status with thyroid volume, goiter, and thyroid nodules [334,335,336,337]. For cardiovascular disease, results are currently conflicting. While some studies have proved the benefits of selenium supplementation on heart and coronary health, especially in males, major side effects (i.e., increased diabetes risk) also appear to be male-specific. Other researchers have found more positive effects among women or no sex association. On the basis of these considerations, selenium metabolism and selenium health effects may differ between females and males, and generalizations should not be made across the sexes [27,146,325,338].

Herein we have reviewed recent results that—although males and females differ considerably with respect to selenium metabolism, selenoprotein expression, and medical selenium effects on health and disease—in most cases did not consider gender as a discriminator. The regulation of selenoprotein expression seems now to be not only tissue-specific and age-related but also sex-specific [27]. The levels of selenoproteins mRNA and selenoproteins in different tissues vary between the sexes with specific expression patterns according to the selenium status, mainly regulated by controlling the translational aspects [339,340]. Additionally, a number of studies on animal models have highlighted that sex hormones also have an impact on the regulation of selenium metabolism and selenoproteins [341]. Thus, an innovative approach that would take into account the concept of “gender medicine” [342,343] should also be considered in the setting of future studies aimed at elucidating the relationship between selenium nutritional status, health or disease state and different male or female gender.

## 15. Conclusions

Selenium is an important microelement involved in a number of biologically essential functions. It is mainly uptaken from the diet (including supplements) and it is incorporated in selenoproteins in the form of selenocysteine. The unique features of the selenol moiety of selenocysteine enable selenoproteins to accomplish a wide variety of different biological functions with respect to their sulfurated analogues. Selenoproteins are indeed involved in several processes, spanning from biosynthesis of hormones to modulation of oxidative stress. Selenoprotein-mediated biochemical mechanisms also play a central role in the prevention, onset, and clinical outcome of a wide number of important diseases which, amongst others, include cancer, diabetes, viral infections (including SARS-CoV-2 and HIV), mental and neurological disorders.

Several studies have been carried out in order to elucidate the role of selenium in the prevention and modulation of such pathologies. Herein, we have reviewed these studies, mainly focusing on the last ten years of research. According to the reported data, a positive relationship between selenium status in the body and a favourable prognosis of the abovementioned pathologies has been generally observed. Furthermore, an adequate intake of selenium (from the diet and/or supplements) is crucially involved in the prevention of an array of diseases, particularly those related to thyroid function, fertility and reproduction, skeletal health, inflammatory based diseases and some mental disorders. An optimal baseline serum selenium status has also been suggested to be a favourable prognostic factor for patients with increased risk of developing severe forms of COVID-19. Preliminary supplementation trials have also shown that selenium could be a therapeutic strategy against the SARS-CoV-2 pandemic that we are still facing. In this regard the selenium therapeutic potential has also been showcased by the fact that ebselen, a selenium-containing small molecule, exhibited remarkable M^pro^ inhibitor properties.

Both housekeeping and stress-related selenoproteins have a role in the abovementioned conditions and their expression is finely regulated according to the selenium concentration in the different cells and systems of the human body. Maintaining adequate levels of selenium in the body is therefore essential to properly support the biological functions of selenoproteins (i.e., antioxidant and immune functions). Nevertheless, there is no reason to supplement selenium over a certain plasma concentration.

The over-expression of some selenoproteins that is achieved through genetic manipulation in animal models is a useful tool for studying the mechanisms of their functioning. However, as discussed above, a selenoenzymes expression higher than normal physiological levels can also occur in some districts of the body as a consequence of the chronic intake of supra-nutritional levels of selenium, with negative outcomes. A potential link between supra-nutritional exposure to selenium in patients with already optimal status at baseline, and an increased risk of developing some pathologies, has been suggested. For example, a similar behaviour has been observed for some cancers, T2 diabetes, Alzheimer’s disease, Parkinson’s disease, and cardiovascular diseases.

The selenium supplementation at supra-nutritional levels may be useful, for short periods or in subjects greatly deficient at baseline, or for patients whose pathologies favour a massive depletion of antioxidants that can aggravate the course of the disease. For example, this has been suggested in the case of some inflammatory or infectious diseases.

According to these considerations a U-shape for the relationship between selenium status and the onset of disease status could be considered. Excessive levels of circulating selenium, deriving from a supra-nutritional intake, could also afford an excessive production of H_2_O_2_ via SEBP1, up to blocking the mitochondrial reactions with consequent cell apoptosis [13,19]. Meanwhile, a high content of selenium in the body pool may result in a dysregulation of tissue-specific selenoprotein expression, with increased levels of antioxidant enzymes with respect to physiological ones. In this way an “overquenching” of ROS—particularly H_2_O_2_—might occur, with the direct consequence of the interruption of some pivotal intracellular signalling pathways relying on ROS, for example insulin secretion [321,344,345,346]. Hydrogen peroxide and superoxide are key species for intracellular signalling pathways. They are produced through controlled mechanisms by more than 40 enzymes, including the NADPH oxidases and the mitochondrial electron transport chain complexes. At physiological levels (in a nanomolar concentration range), H_2_O_2_ takes part in the metabolic regulation and the cellular adaptation to stress. Acting similarly to other reactive species such as nitric oxide and hydrogen sulphide. The physiological oxidative state essential in redox signalling is indeed called *oxidative eustress*. Supraphysiological concentration of reactive species (i.e., H_2_O_2_ roughly above 100 nM), leading to specific oxidation of proteins, altered response patterns, and damage to biomolecules, denote a status called oxidative distress. Selenoproteins are also crucially involved in the maintenance of redox homeostasis [347].

A selenium deficiency can correspond to a reduced general synthesis of selenoproteins, in particular those more strictly dependent on the selenium intake including, but not restricted to, GPXs and TRxRs. This may correspond to an increased oxidative stress and its consequences in the body—such as inflammation and alteration of redox homeostasis—but also in misled protein folding and degradation, impaired protein function, compromised selenium transport to strictly Se-dependent tissues, interactions in calcium signalling pathways, and others, with relevant and severe consequences on the state of health or disease.

A low selenium intake leads to a selenium concentration below the levels for optimum expression of both GPx and SelP. The expression of these selenoproteins is reported to be dependent on the dietary intake of selenium. Several studies report that selenium supplementation (supra-nutritional levels) is effective in enhancing selenium concentration levels. Serum selenium concentrations achieved with supplementation would lead to a maximised/saturated expression of selenoproteins [118,138]. The role of high selenium dietary levels (or selenium supplementation) in enhancing the expression of selenoproteins is well documented in the literature [135,187]. Several other studies describe how the selenium supplementation—and, generally, a high selenium intake (above 60–80 µg/day)—is related to an upregulation of some selenoproteins at their maximum levels. Selenium supplementation-induced upregulation of selenoproteins could occur through a process ascribed either to the reversal of a nutritional deficiency of the element or to a compensatory response towards the pro-oxidant effects of selenium, with both mechanisms potentially operating [316]. Selenium species by themselves and through excess expression of selenoproteins may have adverse effects of potential interest in diabetogenesis [309,314,316]. Potential adverse effects of selenoproteins on the onset of chronic diseases have also been demonstrated in studies involving animal models where selenoproteins levels have been increased by genetic manipulation [34,318,320,321,344] or by injections of purified human Selenoprotein P [324].

In this context, ensuring *optimal* levels of selenoproteins plays an important role in the maintenance of redox homeostasis. According Jablonska and Vinceti [34], fundamental biological evidence should define the exact meaning of *optimal* activity/expression of selenoproteins; particular attention should be devoted to specify whether *optimal* is equal to *maximal*. Thus, it is important to evaluate if the activity/expression of a selenoprotein can be correctly defined as *sub-optimal* only because it is not *maximal* and increased by selenium supplementation. The approach leading to selenium supplementation in order to maximise levels of GPx and SelP should be critically analysed and evaluated. Such an approach relies on the hypothesis that any level of these selenoproteins located below the maximal one is related to Se deficiency and potentially inappropriate for human health protection.

Besides the nutritional effect, selenium-induced upregulation of redox responsive selenoproteins may also be a result of selenium pro-oxidant activity. Thus, in order to provide the ability to elicit an adaptive, compensative response in the presence of oxidant stressors, selenoproteins should not be maximised under normal conditions. On the other hand, laboratory studies suggest that selenium-induced selenoprotein activity is, at least partially, an adaptive response to selenium excess and, potentially, to its toxicity.

However, while selenoproteins may account for some of the effects of treatments with slightly supra-nutritional doses of selenium, other mechanisms—also related to the pro-oxidant properties of selenium—reasonably take place when high supra-nutritional doses of selenium supplements are used. Such mechanisms can also be exploited to develop new potential therapeutic tools.

Additional studies are reasonably needed in order to elucidate all these points, and provide quantitative parameters. Different results have often been reported for men and women with regard to the effects of selenium on health. For this reason, future studies should consider sex differences both in the experimental design and in the analyses of the data.

On the basis of all these considerations, a deep comprehension of the biological function of all selenoproteins, as well as the development of selenium containing small molecules as supplements or drug candidates, represent additional challenges ahead in the field of selenium chemistry, biochemistry, and biology.

## Supplementary Materials

The following are available online at https://www.mdpi.com/article/10.3390/antiox11020251/s1, Table S1: Recent research on cardiovascular diseases—A synthetic overview, Table S2: Recent research on Alzheimer’s diseases—A synthetic overview, Table S3: Recent research on Parkinson’s diseases–A synthetic overview, Table S4: Recent research on Type-2 diabetes—A synthetic overview.
