# Supplementary material for: The Role of Selenium in Pathologies: An Updated Review
Source: Antioxidants (Basel). 2022 Jan 27;11(2):251. doi: 10.3390/antiox11020251 (PMC8868242; doi:10.3390/antiox11020251)
Supplement: Supplementary file 1 [file antioxidants-11-00251-s001.zip › antioxidants-1498410-supplementary.pdf]

ELECTRONIC SUPPLEMENTARY MATERIAL
FOR:

The role of selenium in pathologies: an updated review

Giulia Barchielli, Antonella Capperucci and Damiano Tanini*

Dipartimento di Chimica 'Ugo Schiff', Via della Lastruccia 3-13, 50019 Sesto Fiorentino (FI), Italy; giulia.barchielli1@stud.unifi.it (GB); ntonella.capperucci@unifi.it (A.C.)
* Correspondence: damiano.tanini@unifi.it (D.T.); Tel.: +39-055-4573552

Abstract: Selenium is an essential microelement required for a number of biological functions. Selenium – and more specifically the amino acid selenocysteine – is present in at least 25 human selenoproteins involved in a wide variety of essential biological functions, ranging from the regulation of Reactive Oxygen Species (ROS) concentration to the biosynthesis of hormones. These processes also play a central role in preventing and modulating the clinical outcome of several diseases, including cancer, diabetes, Alzheimer's disease, mental disorders, cardiovascular disorders, fertility impairments, inflammation, and infections (among which SARS-CoV-2). Over the past years, a number of studies focusing on the relationship between selenium and such pathologies have been reported. Generally, an adequate selenium nutritional state – and in some cases selenium supplementation – have been related with improved prognostic outcome and with reduced risk to develop several diseases. On the other hand, supranutritional levels might have adverse effects. The results of recent studies focusing on these topics are summarized and discussed in this review, with particular emphasis to advances achieved in the last decade.

Table of Contents:

Tables S1 *S2*
Tables S2 *S6*
Tables S3 *S8*
Tables S4 *S8*

Table S1. Recent researches on cardiovascular diseases – A synthetic overview

REFERENCE	YEAR	STUDY	AIM	POPULATION *(for meta-analysis the number of considered studied is reported)*	SUPPLEMENT (for trials)	DURATION	MAIN RESULTS
Alehagen, U. *et al.* Ref. 143	2021	Randomized Controlled Trial	Evaluate the effect of a combined supplementation of Selenium and CoQ_{10} on the concentration of D-dimer, a marker associated with cardiovascular mortality	213 subjects aged 70–88 years from a rural municipality in Sweden	Selenium yeast (200 µg/day) and coenzyme Q10 (200 mg/day) (n = 106) or placebo (n = 107)	The follow-up time was 4.9 years	The individuals with a D-dimer level above median at baseline, showed a significant benefit from supplementation, resulting in lower cardiovascular mortality compared to the placebo group.
Al-Taesh, H. *et al.* Ref. 155	2021	Case-control study	Investigate the relationship between aortic valve sclerosis (AVSc) and serum levels of selenium and other minerals.	40 subjects at the Gaziantep University General Hospital (Turkey) with AVSc and a control group with the same gender ratio	//	//	The patients group showed lower serum Se concentrations compared with the healthy group.
Hu, H, *et al.* Ref. 154	2021	Case-control study	Investigate the association between baseline plasma selenium and first stroke risk.	1255 first stroke cases and 1255 matched controls from the Rongcheng County, Shandong Province, and the Lianyungang, Jiangsu Province, (China)	//	//	A significant, non-linear, negative association between plasma selenium and first stroke was found in males but not in females.

Bomer, N. *et al.* Ref. 131	2020	Observational study	Investigate the association between selenium deficiency and heart failure symptoms and outcome.	2516 patients with worsening heart failure from the BIOSTAT-CHF cohort icnluding 69 centres in 11 European countries (The Netherlands, Norway, Sweden, UK, Germany, Poland, Serbia, Slovenia, France, Greece, Italy).	//	//	Selenium deficiency (<70 μg/L in serum) was independently associated with impaired exercise tolerance and a 50% higher mortality rate.
Li, J *et al.* Ref. 130	2020	Observational study	Investigate the relationship between serum selenium and all-cause mortality and cardiovascular disease (CVD) mortality, and the potential gender differences.	2,903 subjects (50,7% female) from the NHANES cohort (USA).	//	//	Selenium was significantly associated with all-cause mortality among both males and females.<br>Selenium was significantly associated with CVD mortality only among females.
Mirdamadi, A.*et al.* ref. 153	2019	Case-control study	Compare selenium level in patients with heart failure and healthy individuals.	32 hospitalized patients with heart failure and 32 healthy controls at the Shariati Hospital, Isfahan (Iran)	//	//	Significantly lower serum selenium was found in patients with heart failure in comparison to normal individuals.<br>A significant reverse relationship between selenium level and left ventricular volume and pulmonary artery pressure was found.
Maugeri, A. *et al.* Ref. 152	2019	Cross-sectional analysis	Investigate the relationship between dietary antioxidant intake (evaluated through a composite dietary antioxidant index - CDAI) and the carotid Intima Media Thickness (cIMT), and other CVD risk factors.	894 subjects from the Kardiovize cohort (Czech Republic)	//	//	CDAI negatively associates with cIMT in women.<br>Dietary intake of selenium and other antioxidants negatively correlated with WHR, BFM,creatinine and total cholesterol/HDL ratio, but positively correlatedwith HDL-cholesterol.

Ju, W. *et al.* Ref. 145	2017	Meta-Analysis	Investigate the impact of selenium on CHD.	16 double blinded placebo-controlled trials for a total of total of 43998 participants	Selenium as selenite, selenium yeast, Brazil nuts, L-selenomethionine, alone (6 trials) or in combination with other molecules (10 trials).	8 weeks – 7,2 years	Selenium supplementation decreased serum C-reactive protein (CRP) and increased the GPx level (suggesting a positive effect on the reduction of oxidative stress). No improvement in lipidemia or reduction of the CHD mortality were observed.
Zhang, X. *et al.* Ref. 157	2016	Meta-Analysis	Assess the discrepancies between observational studies and randomized trial evidence about the relationship between selenium and CVD.	16 prospective observational studies (35607 participants) and 16 randomized controlled trials (37572 participants)	From 75µg to 300µg of selenium per day in the form of L-selenomethionine, sodiumselenite and selenizedyeast	2 weeks to 114 months	Significant inverse association between selenium status and CVD risk within a narrow selenium range. Null effect of selenium supplementation on CVD was observed in randomized controlled trials.
Alehagen, U. *et al.* Ref. 137	2015	Randomized Controlled Trial	Analyze cardiovascular mortality for up to 10 years after a 4 years-long supplementation intervention with Selenium and CoQ10.	443 elderly healthy subjects from a rural municipality in Sweden	200 mg/day of coenzyme Q10 capsules and 200 µg/day of organic selenium yeast tablets or a similar placebo.	10-year follow-up	Significantly reduced cardiovascular mortality could be seen in the individuals that were supplemented with selenium and coenzyme Q_{10}.
Rayman, M.P *et al.* Ref. 150	2011	Randomized, placebo-controlled, parallel-group study stratified by age and sex	Investigate the effect of selenium supplementation on plasma lipids.	501 volunteers aged 60 to 74 years from the PRECISE cohort (UK)	High-selenium yeast: (100µg/day, 200µg/day or 300µg/day) or a yeast-based placebo	6 months	Increased plasma selenium concentration. Significant reduction of total and non-HDL plasma cholesterol for low and medium supplementation with respect to the control. Increased HDL cholesterol for high supplementation. The total/HDL cholesterol ratio decreased progressively with increasing selenium dose.
Bleys, J. *et al.* Ref. 156	2009	Cross-sectional study	Evaluate a possible relationship between serum selenium levels and peripheral arterial disease.	2,062 US men and women 40 years of age or older from the NHANES cohort (USA).	//	//	The association between serum selenium levels and the prevalence of peripheral arterial disease was not statistically significant, although a U-shaped relation was suggested.

Bleys,J. *et al.*<br>Ref. 132	2008	Observational study	Evaluate the association between selenium levels and all-cause and cause-specific mortality .	13887 US adults	//	//	No association was found between serum selenium levels and cardiovascular mortality.
Flores-Mateo, G. *et al.*<br>Ref. 144	2006	Meta-Analysis	Perform a meta-analysis on the association of selenium biomarkers with coronary heart disease in observational studies and on the efficacy of selenium supplements in preventing coronary heart disease in randomized trials.	Twenty-five observational studies (14 cohort and 11 case-control studies) and 6 randomized supplementing trials. Total subjects for the supplementing trials: 17766 participants	Selenium alone or in combination with other vitamins or minerals (4 trials). Selenium was as sodium selenite, selenium yeast or not specified. Selenium doses ranged 75µg- 100µg	0,5-7,6 years	Selenium concentrations were inversely associated with coronary heart disease risk in observational studies.<br>Increased Se levels corresponded to a lower incidence of CHD, in particular in subjects with low dietary intake.<br>In subjects who already had an adequate Se-intake overexposure could even cause cardiovascular damage.
Hercberg, S. *et al.*<br>Ref. 148	2005	Clinical Trial	Assess the effects of antioxidant supplementation on changes in the serum lipid profile of adult participants.	12741French adults (7713 females aged 35-60 years and 5028 males aged 45-60 years) from the SU.VI.MAX cohort	A daily capsule (with 120 mg ascorbic acid, 30 mg vitamin E, 6 mg β-carotene, 100 µg selenium, and 20 mg zinc) or a placebo	Median follow up 7,5 years	No effects on total cholesterol were observed in the supplemented group.<br>Increased triglyceride and lowered HDL-cholesterol levels were found among men but not in women.<br>A possible trend towards hyperlipidemia was suggested for women that received the supplement.
Hercberg, S. *et al.*<br>Ref. 147	2004	Clinical Trial	Test the effect of a multivitamin and mineral supplement in reducing the incidence of cancer and ischemic cardiovascular disease in the general population.	13017 French adults (7876 women aged 35-60 years and 5141 men aged 45-60 years) from the SU.VI.MAX cohort	A daily capsule (with 120 mg ascorbic acid, 30 mg vitamin E, 6 mg β-carotene, 100 µg selenium, and 20 mg zinc) or a placebo	Median follow up 7,5 years	No major differences were detected between the groups in ischemic cardiovascular disease incidence.

Table S2. Recent researches on Alzheimer's diseases – A synthetic overview

REFERENCE	YEAR	STUDY	AIM	POPULATION	SUPPLEMENT	DURATION	MAIN RESULTS
Cardoso, B.R *et al*. Ref. 242	2021	Cross-sectional study	Investigate sex differences in the association between selenium concentration in whole blood and cognitive performance.	2016 participants (984 male and 1032 female) ≥ 60 years from the NHANES cohort (USA)	//	//	An association between selenium status and cognitive performance was observed in males but not in females.
Socha, K. *et al*. Ref. 271	2021	Case-control observational study	Estimate selenium and other antioxidants levels in the serum of patients with Alzheimer's disease (AD) in relation to their cognitive functions and dietary habits.	110 patients with early or moderate AD (aged 54-93 years), and 60 healthy controls (aged 52-83 years) from a Polish cohort.	//	//	Dietary habits have a significant impact on the concentration of the assessed parameters in the serum of people with AD.
Koseoglu, E. *et al*. Ref. 266	2021	Case-control observational study	Investigate the possible relationship of Selenium to the diagnosis and pathophysiology of AD.	40 AD patients in different clinical stages and 40 healthy control subjects, living in a similar environment from the area of Kayseri (Turkey)	//	//	There may be an increase in the selenomethionine unregulated pool and a decrease in the selenocysteine regulated pool of selenium in AD.
Varikasuvu, S.R. *et al*. Ref. 267	2019	Meta-analysis	Investigate selenium status in AD brains compared to controls.	14 studies including 40 observations on selenium concentrations in AD and control brain tissues	//	//	Selenium levels were decreased in the temporal, hippocampal, and cortex regions in AD. The maintenance of selenium homeostasis may be a novel therapeutic strategies in AD.
Tamtaji, O. R. *et al*. Ref. 277	2019	Randomized Controlled Trial	Determine the effects of probiotic (containing *Lactobacillus acidophilus*, *Bifidobacterium bifidum*, and *Bifidobacterium longum*) and selenium co-supplementation on cognitive function and metabolic status among patients with AD.	73 AD patients aged 55 to 100 years from Iran	Selenium (200 µg/day) plus probiotic (2 × 109 CFU/day each) (n = 27), selenium (200 µg/day) (n = 26) or placebo (n = 26)	12 weeks	Probiotic and selenium co-supplementation f improved cognitive function and some metabolic profiles in patients with AD.

Vinceti, M. *et al.* Ref. 272	2019	Case-control study	Investigate the relation between selenium in cerebrospinal fluid (CSF) and AD.	56 patients with mild cognitive impairment and 33 patients with established AD from Italy (Modena and Reggio Emilia University Hospital).	//	//	AD risk was inversely correlated with inorganic selenium species and with the organic form bound to selenoprotein P in the CSF. Se-bound to other organo-selenium species was positively correlated with AD risk.
Cardoso, B.R *et al*. Ref. 276	2019	Randomized Controlled Trial	To evaluate selenium and selenoproteins in serum and cerebrospinal fluid (CSF) after a supplementation intervention with selenium at nutritional or supranutritional doses	40 patients recruited from 4 centers in Melbourne, Australia	0.32 mg of sodium selenate, 3/day, 10 mg of sodium selenate 3/day, or a placebo	24 weeks	Sodium selenate supranutrional supplementation induced an increase in selenium uptake into the CNS, with elevation in CSF selenium and corresponding to subtle but significant improvement in MMSE (Mini-Mental Status Examination test) score.
Cardoso, B.R *et al.* Ref. 273	2017	Multicenter observational study	Assessed Se status in different biofluids.	1112 Australian individuals aged over 60 and clinically classified as: healthy controls, mild cognitive impairment (MCI) and AD patients	//	//	No differences in total Se concentration in serum or CSF of AD subjects were observed compared to MCI patients and healthy controls. Se levels in erythrocytes were decreased in AD patients compared to controls.
Cardoso, B.R *et al*. Ref. 241	2016	Randomized controlled trial	Investigate cognitive effects of the consumption of Brazil nuts, considered the best selenium food source.	31 older adults with mild cognitive impairment from Brazil (University of São Paulo Medical School)	One Brazil nut daily (estimated selenium 288.75 µg/day)	6 months	The intake of Brazil nuts restores Se deficiency and can have positive effects on some cognitive functions of older adults with mild cognitive impairment.
Kesse-Guyot, E. *et al.* Ref. 239	2011	Double-blind, placebo- controlled randomized trial	Estimate the long-term effects of antioxidant nutrient supplementation on the cognitive performance.	447 French participants aged 45-60 years from the SU.VI.MAX cohort	A daily capsule (with 120 mg ascorbic acid, 30 mg vitamin E, 6 mg β-carotene, 100 µg selenium, and 20 mg zinc) or a placebo	6 years	This study supports the role of an adequate antioxidant nutrient status in the preservation of verbal memory, in subjects who were non-smokers or who had low serum vitamin C concentrations at baseline.
Loef, M. *et al.* Ref. 236	2011	Systematic review	Investigate the potential role of selenium in AD treatment.	9 placebo-controlled studies, 4 prospective, 4 cross-sectional, 15 case control, 24 autopsies			No evidence for a role of selenium in the treatment of AD, but potential preventive role.
Shahar, A. *et al.* Ref. 240	2010	Observational study	Investigate the association between plasma selenium concentrations and the presence of "soft" neurological signs related to neurodegeneration.	1012 participants of age ≥65 years from the InCHIANTI cohort (Tuscany, Italy)	//	//	Lower selenium levels were significantly associated with decreased performance in neurological tests of coordination.

Gao, S. *et al.*<br>Ref. 238	2007	Observational study	Clarify a possible relationship between selenium levels in the body and cognitive functions.	2000 aged 65 years or older subjects from the rural areas of Sichuan Province and Shandong Province (China).	//	//	Lower selenium levels measured in nail samples were significantly associated with lower cognitive scores.
Meseguer, I. *et al.*<br>Ref. 274	1999	Case-controls study	To evaluate possible differences in serum an cerebrospinal fluid (CSF) selenium levels between AD patients and healthy controls.	27 patients with Alzheimer's disease (AD) and 34 matching controls from Spain	//	//	No significant differences were observed in CSF and serum selenium levels between AD-patient and control groups.

Table S3. Recent researches on Parkinson's diseases – A synthetic overview

REFERENCE	YEAR	STUDY	AIM	POPULATION	MAIN RESULTS
Zhang, Y.J. *et al.*<br>Ref. 287	2021	Meta-analysis	Investigate the correlation between selenium levels and Parkinson's Disease (PD).	12 articles involving 601 PD patients and 749 controls	Cerebrospinal fluid Se levels were significantly higher in PD patients than in controls.
Adani, G. *et al.*<br>Ref. 286	2020	Meta-analysis	Compare the levels of selenium, copper, iron, and zinc in PD patients and controls in blood (whole blood, serum/plasma) or cerebrospinal fluid (CSF).	56 case-control studies	The overexposure in the central nervous system to selenium may be a risk factor of the disease.

TableS4. Recent researches on Type 2 diabetes – A synthetic overview

REFERENCE	YEAR	STUDY	AIM	POPULATION	MAIN RESULTS
Wongdokmai, R. *et al.*<br>Ref. 314	2021	Observational study	Evaluate the relationships of selenium status and inflammation with type 2 diabetes and obesity.	655 Thai subjects from the EGAT2 cohort (Thailand)	Serum selenium status may be linked to both obesity and type 2 diabetes through its effects on signalling pathways.
Vinceti, M. *et al.*<br>Ref. 316	2021	Observational study	Investigate the association between selenium intake and first hospitalization event for type 2 diabetes during a median follow-up period of 8.2 years.	21335 diabetes-free participants in the Moli-sani cohort, Italy	High dietary selenium intake was associated with increased risk of hospitalization for diabetes.
Siddiqi, S.M. *et al.*<br>Ref. 313	2020	Observational study	Investigate the significance of dietary Se with respect to type 2 diabetes..	8824 subject from the Northern China	This research supports the role of high dietary Se in the incidence of T2D.
Liao, X.L. *et al.*<br>Ref. 315	2020	Observational study	Investigate the relationship between circulating selenium and type 2 diabetes (diabetes mellitus).	2903 participants (580 were affected by type 2 diabetes) from the NHANES cohort, USA.	Serum selenium was independently associated with type 2 diabetes only in female aged <65 years.

Jacobs, E.T. *et al*. Ref. 308	2019	Randomized Controlled Trial	Test the hypothesis that selenium supplementation adversely affects pancreatic β-cell function and insulin sensitivity.	400 men and women aged 40 to 80 years recruited from Arizona, Colorado, Texas, and New York that were supplemented with 200 μg/day of selenium for 6 months.	No significant adverse effect of daily Se supplementation with 200 μg/day of selenized yeast on β-cell function or insulin sensitivity was highlighted.
Stranges, S. *et al*. Ref. 317	2019	Randomized Controlled Trial	Investigate the effect of selenium supplementation at different dose levels on changes in HbA1c after 6 months and 2 years in a population of low selenium status.	491 volunteers aged 60 to 74 years from the Denmark PRECISE cohort were supplemented with ith 100, 200 or 300 µg selenium/day as selenium-enriched yeast or placebo-yeast.	Selenium supplementation did not significantly affect HbA1c levels after 2 years in an elderly European population of low selenium status.
Kim, J. *et al*. Ref. 310	2019	Meta-analysis	Clarify the impact of Se on type 2 diabetes.	20 studies for a total of 47930 participants	High levels of selenium were significantly associated with the presence of type 2 diabetes.
Vinceti, M. *et al*. Ref. 309	2018	Meta-analysis	Elucidate the possible dose-response relation between selenium and type 2 diabetes.	18 studies including 13 non-experimental studies and 5 randomized clinical trial	A little increased risk of type 2 diabetes was observed for higher plasma or serum selenium levels.
Kohler, L.N. *et al*. Ref. 311	2018	Meta-analysis	Synthesize the evidence of any association between selenium and type 2 diabetes (T2D).	15 studies including 13 observational studies and 2 randomized clinical trials	Observational studies mainly showed a statistically significant positive association between concentrations of Se and odds for T2D.<br>Randomized clinical trials did not highlight an increased risk deriving from Se supplementation with respect to the placebo.<br>The risk for T2D may vary according the duration of the exposure.
Yang, S.J. *et al*. Ref. 324	2011	Comparative study	Examine the relationship between circulating SelP levels and clinical parameters associated with insulin resistance.	100 subjects with diverse glucose tolerance statuses. From Korea	Serum SelP concentrations were significantly higher in patients with type 2 diabetes or prediabetes.
